# Testing biological network motif significance with exponential random graph models

**DOI:** 10.1007/s41109-021-00434-y

**Published:** 2021-11-22

**Authors:** Alex Stivala, Alessandro Lomi

**Affiliations:** 1 Institute of Computational Science, Università della Svizzera italiana, Via Giuseppe Buffi 13, 6900 Lugano, Switzerland; 2 The University of Exeter Business School, Rennes Drive, Exeter, EX4 4PU UK

**Keywords:** Motif, Biological network, Exponential random graph model, ERGM

## Abstract

Analysis of the structure of biological networks often uses statistical tests to establish the over-representation of motifs, which are thought to be important building blocks of such networks, related to their biological functions. However, there is disagreement as to the statistical significance of these motifs, and there are potential problems with standard methods for estimating this significance. Exponential random graph models (ERGMs) are a class of statistical model that can overcome some of the shortcomings of commonly used methods for testing the statistical significance of motifs. ERGMs were first introduced into the bioinformatics literature over 10 years ago but have had limited application to biological networks, possibly due to the practical difficulty of estimating model parameters. Advances in estimation algorithms now afford analysis of much larger networks in practical time. We illustrate the application of ERGM to both an undirected protein–protein interaction (PPI) network and directed gene regulatory networks. ERGM models indicate over-representation of triangles in the PPI network, and confirm results from previous research as to over-representation of transitive triangles (feed-forward loop) in an *E. coli* and a yeast regulatory network. We also confirm, using ERGMs, previous research showing that under-representation of the cyclic triangle (feedback loop) can be explained as a consequence of other topological features.

**Supplementary Information:**

The online version contains supplementary material available at 10.1007/s41109-021-00434-y.

## Introduction

Molecular interactions in biological systems are often represented as networks (Winterbach et al. 2013). Some such networks are inherently undirected, such as protein–protein interaction (PPI) networks (De Las Rivas and Fontanillo 2010). Others may be directed, such as gene regulatory networks, where nodes represent operons, and arcs (directed edges) represent transcriptional interactions between them. Much research with such biological networks has concerned “motifs”, small subgraphs which occur more frequently than would be expected by chance. Motifs have been considered the building blocks of complex networks (Alon 2007; Ciriello and Guerra 2008; Milo et al. 2002; Shen-Orr et al. 2002). The biological significance of network motifs derives from their possible interpretation as signs of evolutionary events (Middendorf et al. 2005; Rice et al. 2005).

Two simple examples of motifs in undirected networks are triangles (three-cycles) and squares (four-cycles) (Rice et al. 2005). Directed networks allow for a larger set of potentially important motifs (Middendorf et al. 2005; Milo et al. 2002; Rice et al. 2005), which can be quite complicated, leading to problems of consistency in their definition (Konagurthu and Lesk 2008b).

**Fig. 1:**
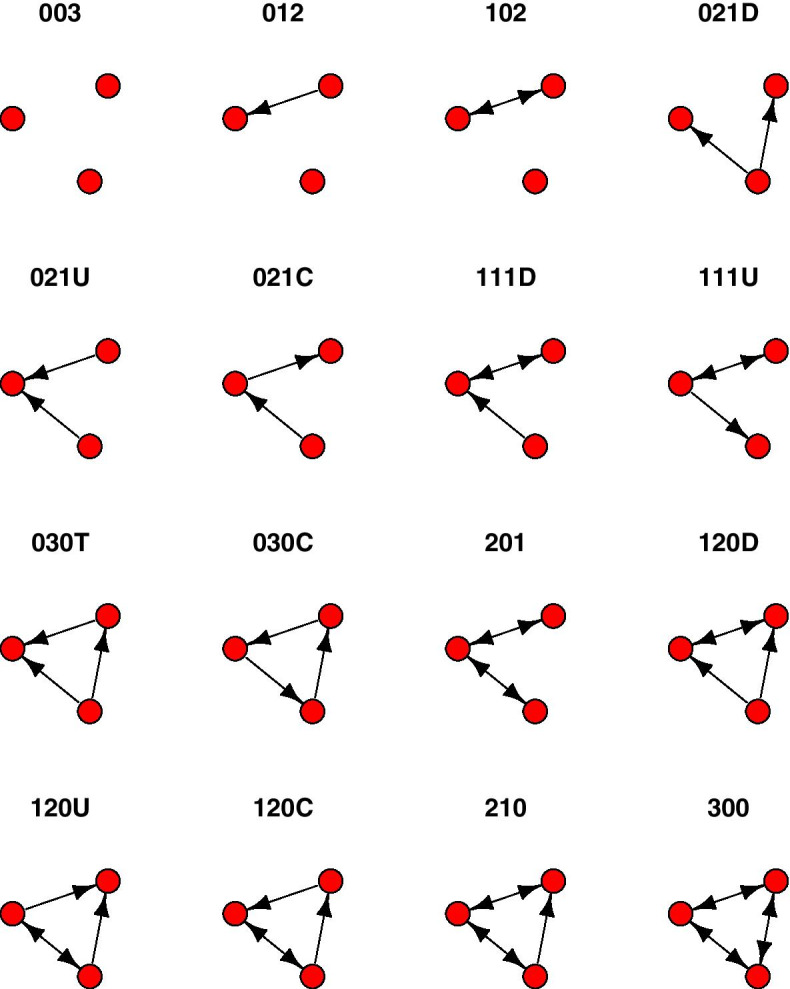
Triad census classes labeled with the MAN (mutual, asymmetric, null) dyad census naming convention. When the dyad census does not uniquely identify a triad, a letter designating “up”, “down”, “transitive”, or “cyclic” is appended

It is worth noting that such (three-node) motifs are an idea with a long history in social network analysis, where the counts of all sixteen possible three-node directed graphs (triads) are known as the triad census (Davis and Leinhardt 1967; Holland and Leinhardt 1970, 1976; Wasserman and Faust 1994). A systematic naming convention has been developed that is based on the number of mutual, asymmetric, and null (*M*, *A*, and *N*) dyads in the triad, followed by a letter to distinguish the orientation if it is not unique (Fig. 1). For example, the transitive triangle is designated 030T, which distinguishes it from the cyclic triad 030C. Although in common usage in social network research, and cited by Milo et al. (2002) and Saul and Filkov (2007) in the context of biological networks, this naming convention is rarely used in discussions of motifs in the bioinformatics or biology literature. There are efficient algorithms for computing the triad census (Batagelj and Mrvar 2001; Moody 1998), implemented in widely used general purpose graph libraries such as igraph (Csárdi and Nepusz 2006) and NetworkX (Hagberg et al. 2008). The triad census has recently been extended to colored triads, that is, distinguishing the nodes in the triads based on a categorical attribute assigned to them (Lienert et al. 2019). It has long been noted in the social networks literature that the dyad census constrains the triad census, and yet empirical social networks often still have counts for some triads greater than expected given those constraints (Faust 2010).

To determine if a motif is over-represented, the count of the motif in an observed network is compared to the distribution of its counts in a set of simulated random networks (Ciriello and Guerra 2008) (it is also possible to determine the significance of motif over-representation without simulation (Martorana et al. 2020; Picard et al. 2008)). This leads to the problem of choosing the appropriate random networks (null model), and some supposed motifs have been found to not be significantly over-represented, and occur with the observed frequencies simply due to topological properties of random networks (Konagurthu and Lesk 2008a) or correlations between motifs created by the randomization process (Ginoza and Mugler 2010), although such correlations can also occur even with uniform sampling (Fodor et al. 2020).

Estimating motif (triad census) significance by comparing the triad census of an empirical network to that of ensembles of random graphs also has a long history, for example the conditional uniform graph (CUG) distribution (Anderson et al. 1999; Butts 2008; Mayhew 1984), conditional on the dyad census (U|MAN) (Holland and Leinhardt 1976), or on the degree distribution (Snijders 1991). A more modern variation on a similar idea is the *dk*-series (Mahadevan et al. 2006; Orsini et al. 2015), a sequence of nested network distributions of increasing complexity, fitting in turn density, degree distribution, degree homophily, average local clustering, and clustering by degree (Orsini et al. 2015).

The recent work of Fodor et al. (2020) shows that the assumptions of mainstream methods for motif identification, specifically normally distributed motif frequencies and independence of motifs, do not always hold, and that, as a consequence, such methods cannot always correctly estimate the statistical significance of motif over-representation.

Aside from such intrinsic statistical limitations, it may be the case that the apparent statistical over-representation of motifs has no evolutionary or functional significance (Ingram et al. 2006; Mazurie et al. 2005; Payne and Wagner 2015), and the choice of null model is a critical factor in this lack of evident relationship between over-representation and evolutionary preservation (Beber et al. 2012; Mazurie et al. 2005). Alternatively, the apparent lack of functional significance (Payne and Wagner 2015) may be due to too narrow a definition of “function” (Ahnert and Fink 2016). Recently, it has also been suggested that elementary motifs are a lower level of structure than that which is most functionally relevant in gene regulatory networks characterizing different physiological states (Lesk and Konagurthu 2021).

It might also be the case that particular motifs are over-represented, not because they are evolutionarily selected for function, but because of spatial clustering (Artzy-Randrup et al. 2004). For example, in the context of PPI networks, we might expect that interactions would be over-represented between proteins that share a subcellular location, and under-represented between those that do not, since proteins known to interact usually have the same subcellular locations (von Mering et al. 2002). Indeed PPI networks can be used as predictors of subcellular location (Kumar and Ranganathan 2010; Shin et al. 2009).

There are many algorithms for motif discovery in complex networks; for recent reviews, see Jazayeri and Yang (2020), Patra and Mohapatra (2020) and Yu et al. (2020). In the present work we are considering only static, not temporal, networks. Although they differ in many details, especially regarding computational efficiency and scalability, these motif discovery algorithms work fundamentally in the manner described above. That is, they count occurrences of a motif in the observed network, and compare this to the distribution of the motif’s frequency in an ensemble of randomized versions of the original network (typically preserving degree sequence). Therefore these conventional methods all test the significance of one motif at a time, assuming independence of motifs, and are all potentially subject to the problems described by the recent work of Fodor et al. (2020), mentioned above. That is, that the assumptions of independence and normal distribution of motif frequencies may not hold, and that therefore these methods might not be able to correctly estimate the statistical significance of motif over-representation.

In this work we describe a different approach to determining motif significance in complex networks, which can potentially overcome these problems. Rather than comparing the observed frequency of a candidate motif to its frequency in a set of randomized networks, we take a model-based approach. Specifically, we estimate parameters of a model (an exponential random graph model, abbreviated ERGM) of the observed network. These parameters correspond to substructures which resemble potential motifs of interest. This allows the significance of the candidate motifs to be tested simultaneously in a single model, in such a way that independence of the motifs is not assumed.

Once such a model is estimated, it can also be used to test for motif significance in the traditional way, using the ERGM to simulate an ensemble of random networks. Recently, this approach was used test for motifs (dyads, triads, and tetrads; that is, two, three, and four node motifs) in a collection of social (rather than biological) networks (Felmlee et al. 2021). Using ERGM rather than degree-preserving randomization, “reduces the scope for misleading results by controlling for multiple, potential correlates in the same set of random models.” (Felmlee et al. 2021, p. 2).

We demonstrate the ERGM approach in biological networks (both undirected (PPI) and directed gene regulatory networks) using some recently developed ERGM estimation methods (Borisenko et al. 2019; Byshkin et al. 2016, 2018; Stivala et al. 2020), which allow estimation of models for larger networks than was practical with earlier methods of ERGM parameter estimation.

The remainder of this article is organized as follows. First, we describe ERGMs, and review the literature on the application of ERGMs to biological networks. We then report the biological networks considered in this work, and the details of the ERGM configurations, estimation methods, and goodness-of-fit tests we used. Following that, we present and discuss new ERGM models of these networks, comparing the inferences as to motif significance with existing published results using conventional motif discovery methods. In the next section, we detail the limitations of this application of ERGMs, and indicate some potential future work. We conclude with a summary of the inferences drawn from the ERGM models of the networks considered.

## Exponential random graph models

ERGMs are widely used in the social sciences, typically to model social networks (Amati et al. 2018; Koskinen 2020; Lusher et al. 2013; Robins et al. 2007a). Cimini et al. (2019) is a recent review of ERGMs for modeling real-world networks, from a statistical physics viewpoint.

An ERGM is a probability distribution with the form

1 $$\begin{aligned} \Pr (X =x) = \frac{1}{\kappa (\theta )}\exp \left( \sum _A \theta _A z_A(x)\right) \end{aligned}$$

where

- $X = [X_{ij}]$ is a 0–1 matrix of random tie variables,
- *x* is a realization of *X*,
- *A* is a “configuration”, a (small) set of nodes and a subset of ties between them,
- $z_A(x)$ is the network statistic for configuration *A*,
- $\theta _A$ is a model parameter corresponding to configuration *A*,
- $\kappa (\theta )$ is a normalizing constant to ensure a proper distribution.

Given an observed network *x*, we aim to find the parameter vector $\theta$ which maximizes the probability of *x* under the model. Then for each configuration *A* in the model, its corresponding parameter $\theta _A$ and its estimated standard error allow us to make inferences about the over- or under-representation of that configuration in the observed network. If $\theta _A$ is significantly different from zero, then if $\theta _A > 0$ the configuration *A* is over-represented, or under-represented if $\theta _A < 0$.

**Fig. 2:**
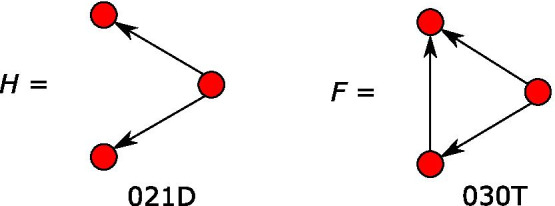
Motif examples. *F*, the transitive triangle (triad 030T) is not a special case of *H*, the out-star (triad 021D), when considered as motifs (or triad census classes): they are distinct induced subgraphs of three nodes. However, when considered as ERGM configurations, since *H* is a subgraph (but not an induced subgraph) of *F* (the transitive triangle is formed by “closing” the out-star with an additional arc), in their corresponding statistics both *F* and *H* are counted for an occurrence of *F*

Note that a “configuration”, unlike a motif (in its most common usage) or the triad census classes, is not an induced subgraph. That is, it does not include every edge in the original graph of which it is a subgraph: a configuration is any occurrence of the substructure in question in the graph; it is defined only by its edges, not by its edges and non-edges. See Fig. 2 for an example based on one from Fodor et al. (2020, Fig. 5B).

ERGMs solve the problem of the need to correct for correlations between motif occurrences, and also other attributes such as subcellular location (functional and evolutionary significance is another matter entirely). Given an observed network, model parameters can be estimated by maximum likelihood. Hence parameters corresponding to candidate motifs such as triangles can be estimated, and a positive significant parameter would indicate triangles occurring more frequently than by chance, *given the other parameters in the model* (which would include parameters to control for density and degree distribution, for example). ERGMs allow different structural configurations to be incorporated, as well as configurations based on node attributes (such as physico-chemical properties, or spatial locality), and the significance of the configurations can then be assessed given all the other structural and other configurations included in the model.

ERGMs fulfill all of the desirable criteria for improved network models listed by de Silva and Stumpf (2005, p. 427). They take into account that networks are finite. Indeed, far from requiring very large networks to fit the requirements of mean-field theories, they are dependent on network size and do not scale consistently to infinity (Rolls et al. 2013; Schweinberger et al. 2020; Shalizi and Rinaldo 2013)—a property that can be used to estimate population size from network samples (Rolls and Robins 2017). They can handle modular organization or community or block structure (Babkin et al. 2020; Fronczak et al. 2013; Gross et al. 2021; Schweinberger 2020; Schweinberger and Handcock 2015; Schweinberger and Luna 2018; Wang et al. 2019), samples from larger networks (An 2016; Handcock and Gile 2010; Pattison et al. 2013; Stivala et al. 2016), and missing data (Koskinen et al. 2013; Robins et al. 2004). And finally, they are flexible at incorporating additional information such as nodal attributes, including dyadic attributes, such as distances between nodes. ERGMs have also been extended to handle valued networks (Desmarais and Cranmer 2012; Krivitsky 2012) and dynamic (time-varying) networks (Krivitsky and Handcock 2014), and to use graphlets (Pržulj 2007) as the ERGM configurations (Yaveroǧlu et al. 2015).

Despite these potential advantages, however, ERGM parameter estimation is a computationally intractable problem, and in practice it is generally necessary to use Markov chain Monte Carlo (MCMC) methods (Hunter et al. 2012). A variety of algorithms for ERGM model fitting (Hummel et al. 2012; Hunter and Handcock 2006; Krivitsky 2017; Snijders 2002) are implemented in widely used software packages such as statnet (Handcock et al. 2008; Hunter et al. 2008; Morris et al. 2008) and PNet/MPNet (Wang et al. 2009), and Bayesian methods are also available (Caimo and Friel 2011, 2014). These packages also implement the so-called “alternating” or “geometrically weighted” configurations (Robins et al. 2007b; Snijders et al. 2006), which alleviate problems with model “near-degeneracy”, where the model’s probability mass is concentrated in a very small region of possible networks, which can occur when only simple configurations, such as stars and triangles, are used (Hunter et al. 2012).

Until recently, the computational difficulty of ERGM parameter estimation has limited its application to biological networks, which are often larger than the social networks (traditionally measured by observations and surveys, rather than online social networks) for which the techniques were developed. Now, however, advances such as snowball sampling and conditional estimation (Pattison et al. 2013; Stivala et al. 2016), improved ERGM distribution samplers such as the “improved fixed density” (IFD) sampler (Byshkin et al. 2016), and new estimation algorithms (Hummel et al. 2012), including the “Equilibrium Expectation” (EE) algorithm (Byshkin et al. 2018; Borisenko et al. 2019) and its implementation for large directed networks (Stivala et al. 2020), have reduced by orders of magnitude the time taken to estimate ERGM parameters.

## Literature review of application of ERGMs to biological networks

ERGMs were first applied to biological networks by Saul and Filkov (2007), who estimated model parameters for *Escherichia coli* (Salgado et al. 2001) and yeast regulatory networks, and a collection of metabolic networks. As well as introducing the use of ERGMs to the field of bioinformatics for analyzing biological networks, Saul and Filkov (2007) used ERGM models to build topological profiles which they showed to be capable of classifying organisms into biological and functional groups. With the algorithms and implementations available at the time, the larger networks could only be estimated by maximum pseudo-likelihood (Strauss and Ikeda 1990), an approximation which is now considered problematic (van Duijn et al. 2009; Hunter et al. 2012; Robins et al. 2007b) and useful mostly for obtaining initial parameter estimates for a more accurate (but also more computationally expensive) method (Hummel et al. 2012; Hunter and Handcock 2006; Krivitsky 2017). Further, all the networks in Saul and Filkov (2007) were treated as undirected, thereby losing important directional information (and not, for example, being able to distinguish between cyclic and transitive triads) in regulatory networks. The *E. coli* regulatory network, treated as undirected, was also used as an example application of the “stepping” algorithm for ERGM estimation by Hummel et al. (2012).

Exponential random graph models for similar *E. coli* regulatory networks were described by Begum et al. (2014), leaving the networks directed rather than treating them as undirected. These models were very simple, however, including only Arc and In-star terms, and therefore model degree distribution, but not triangular motifs.

Bayesian estimation of an ERGM model of a human PPI network with 401 proteins was described by Bulashevska et al. (2010). This model used only very basic structural features (not including any triangular structures, for example), but made use of nodal attributes, specifically a binary variable indicating if the protein is disordered. This ERGM was not used to analyze network motifs, but rather the relationship between disordered proteins and their “sociality”, a measure of their importance in the PPI network, finding that intrinsically disordered proteins tend to be more “social” (Bulashevska et al. 2010). In their Conclusions, Bulashevska et al. (2010) suggest that “The ERGM modelling of networks offers a natural way of assessing importance of the network motifs” (Bulashevska et al. 2010, p. 13).

Similar techniques, that is, Bayesian estimation of ERGMs with only very simple structural terms, have also been used with gene–gene relationship networks to model mechanisms of gene dysregulation (Azad et al. 2017). These models were used to infer potential aberrant gene pairs, and suggested a novel pattern of aberrant signaling (Azad et al. 2017).

A mixture ERGM was introduced by Wang et al. (2019) and applied to a yeast gene interaction network with 424 genes (Schuldiner et al. 2005; Wang et al. 2019). The model included geometrically weighted in-degree and out-degree terms, but not any triangular terms; the interest is rather in the clusters it finds, which may be used to predict function (Wang et al. 2019).

An ERGM incorporating a directed form of the degree-corrected stochastic blockmodel (Karrer and Newman 2011) was introduced by Gross et al. (2021), and applied to the connectome of the *C. elegans* worm (279 nodes representing neurons), and an *A. thaliana* PPI network (4344 nodes representing proteins). These models assume dyadic independence, and hence triangular configurations could not be incorporated. The advantage of the mixture ERGM (Wang et al. 2019) or stochastic blockmodel ERGM generalizations ($\beta$-SBM and $p_1$-SBM (Gross et al. 2021)) is that they can capture heterogeneity in clusters found in the network, but we do not address cluster or community structure here.

ERGMs have been applied to neural networks with 90 nodes, representing brain regions (Simpson et al. 2011, 2012), finding that an ERGM approach outperforms conventional approaches for constructing group-based representative brain networks (Simpson et al. 2012). Bayesian ERGM techniques, with 96 nodes representing brain regions, have been used to model brain networks over the human lifespan (Sinke et al. 2016). Recently, Bayesian ERGMs, extended to multiple networks, were used to compare functional connectivity structure across groups of individuals (Lehmann et al. 2021).

ERGMs have also been used to model human brain networks inferred from electroencephalographic (EEG) signals; these networks have 56 (the number of EEG sensors) nodes (Obando and De Vico Fallani 2017). These models showed that clustering and node centrality (as reflected by over-representation of triangles and stars) better explained global properties of the brain networks than other graph metrics, supporting the view that segregated modules exchange information via hubs.

An enhanced version of the generalized (or valued) ERGM (Desmarais and Cranmer 2012) was used to model the human Default Mode Network (DMN) with 20 nodes, representing brain regions (Stillman et al. 2017). This model showed that the DMN appears to be organized in a “segregated highway” structure, that is, with fewer hubs and more triadic closure than expected, in contrast to “small world” structure of the whole-brain network (Stillman et al. 2017). This work is an example of an ERGM that incorporates spatial distances, in the form of three-dimensional Euclidean distances between nodes.

A Bayesian ERGM has been used to model transient structure in intrinsically disordered proteins, providing a means for identifying transient structures that differ in favorability across variants (Grazioli et al. 2019a). A specific family of ERGMs has been used to model amyloid fibril topologies, leading to the construction of a systemic nomenclature that can classify all known amyloid fibril structures, and a simulation technique that can explore the kinetics of fibril self-assembly (Grazioli et al. ﻿2019b).

Simple ERGMs for undirected networks (*A. thaliana*, yeast, human, and *C. elegans* PPI networks, and undirected versions of *E. coli* regulatory and *Drosophila* optic medulla networks) were estimated in Byshkin et al. (2018, S.I.), demonstrating that the EE algorithm could be used to estimate in minutes a model that takes many hours or is practically impossible with earlier methods. In addition, a more complex model of the *A. thaliana* PPI network was estimated, showing not just the over-representation of the triangle motif, but also the tendency for plant-specific proteins to interact preferentially with each other, and for kinases to interact preferentially with phosphorylated proteins (Byshkin et al. 2018). However that work dealt only with undirected networks. An implementation of the EE algorithm for directed networks was described in Stivala et al. (2020), but no biological networks were considered in that work.

## Methods

### Network data

We obtained a yeast PPI network (von Mering et al. 2002) from the igraph (Csárdi and Nepusz 2006) Nexus network repository (this is no longer available, we used the network downloaded on 10 November 2016). The yeast PPI network has the proteins annotated with one of 12 functional categories (Mewes et al. 2002; Ruepp et al. 2004) (or “uncharacterized”), as described in the Supplementary Information of von Mering et al. (2002).

We obtained a human PPI network from the HIPPIE database (Alanis-Lobato et al. 2016; Schaefer et al. 2012, 2013; Suratanee et al. 2014), version 2.2, downloaded from http://cbdm.uni-mainz.de/hippie/ (accessed 12 June 2021). Edges in this network are labeled with a confidence score between zero and one. We built a binary “high confidence” network by selecting edges where the score is $\ge 0.70$, the third quartile of the score distribution.

To annotate nodes in the human PPI network with their subcellular location using terms in the Gene Ontology (GO) (Ashburner et al. 2000), we used the Protein ANalysis THrough Evolutionary Relationships (PANTHER) database (Mi et al. 2019, 2021). We used the PANTHER database version 16.0 downloaded from http://data.pantherdb.org/ftp/sequence_classifications/current_release/PANTHER_Sequence_Classification_files/PTHR16.0_human (accessed 21 June 2021). We used the R package GOxploreR (Manjang et al. 2020, 2021) to rank the GO terms for subcellular component in the PANTHER database, and annotated each node (representing a protein) in the network with the highest ranking term for that protein. This results in a cellular component GO term for 6131 of the 11,517 nodes (53%) in the human PPI network. The cellular component GO terms are treated as a categorical attribute, of which there are 271 unique values in the data. The nodes with no cellular component GO term assigned are given an “NA” category, which, when used in the “Match” statistic in ERGM modeling, does not match any category (including the NA category itself).

The previously mentioned *E. coli* regulatory network (Salgado et al. 2001; Shen-Orr et al. 2002) was obtained via the statnet package (Handcock et al. 2008, 2016). Following Hummel et al. (2012), we removed the loops (self-edges) representing self-regulation, and considered self-regulation instead in a simplistic way by a binary node attribute designated “self” which is true when a self-loop was present and false otherwise. In some models, we use the original version of this network with self-edges retained, and when this is done it is noted in the results. We also obtained a *Saccharomyces cerevisiae* (yeast) regulatory network (Costanzo et al. 2001; Milo et al. 2002) (http://www.weizmann.ac.il/mcb/UriAlon/download/collection-complex-networks; accessed 29 April 2019) and processed it in the same way.

For all networks, we removed multiple edges and, unless noted otherwise, self-loops, where these are present.

**Fig. 3:**
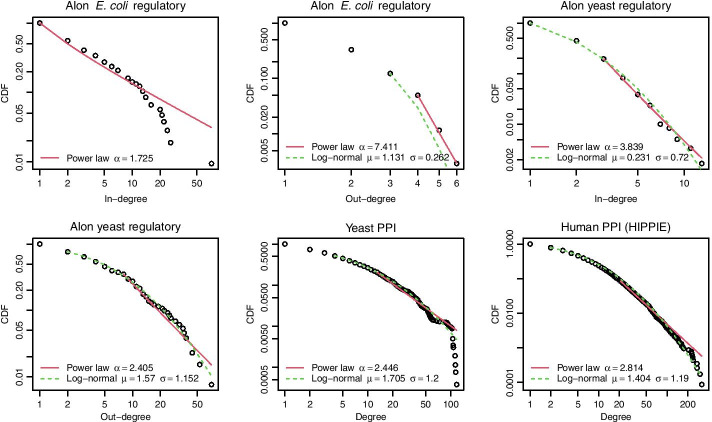
Degree distributions of the networks. Power law and log-normal distributions fitted to the CDF for degree distributions of the networks (in- and out-degree for directed networks, degree for undirected networks). All distributions apart from the *E. coli* in-degree distribution (for which a log-normal distribution could not be fitted), and the Human PPI (HIPPIE) degree distribution (which is not consistent with a power law distribution, $p < 0.01$), are consistent with both power law and log-normal distributions

Summary statistics of the networks are in Table 1 and the degree distributions of the networks are shown in Fig. 3. In this figure, $\alpha$ is the exponent in the discrete power law distribution $\Pr (X=x) = Cx^{-\alpha }$ (where *C* is a normalization constant), and $\mu$ and $\sigma$ are the parameters (respectively, mean and standard deviation of $\log (x)$) of the discrete log-normal distribution. Power law and log-normal distributions were fitted using the methods of Clauset et al. (2009) implemented in the poweRlaw package (Gillespie 2015).

**Table 1:** Summary statistics for the biological networks

Network	Directed	Nodes	Edges	Density	Clustering coefficient
Yeast PPI	No	2617	11855	0.00346	0.46862
Human PPI (HIPPIE)	No	11517	47184	0.00071	0.03765
Alon *E. coli* regulatory	Yes	423	519	0.00291	0.02382
Alon yeast regulatory	Yes	688	1079	0.00228	0.01625

“Clustering coefficient” is the global clustering coefficient (transitivity)

### ERGM configurations

**Fig. 4:**
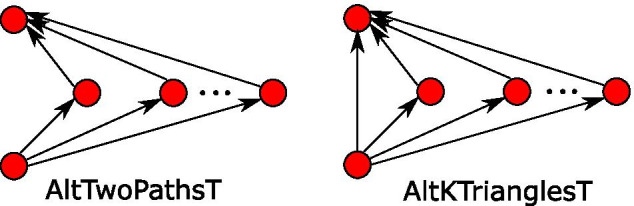
Alternating two-paths and alternating transitive triangles ERGM configurations for directed networks. Unlike motifs, ERGM configurations are not induced subgraphs, so it is normal (and often required) for one to be a subgraph of another. So AltTwoPathsT and AltKTrianglesT are frequently included in a model together, with AltKTrianglesT consisting of the AltTwoPathsT configuration “closed” by the addition of an arc

The ERGM parameters used in the models for undirected networks are shown in Table 2, and those for directed networks in Table 3. Detailed descriptions of these parameters and their corresponding statistics can be found in Lusher et al. (2013); Robins et al. (2007a, 2007b, 2009); Snijders et al. (2006); Stivala et al. (2020), but two of the important ones used in this work are shown in Fig. 4.

**Table 2:** Parameters for undirected networks

Effect	Description
Edge	Baseline density
A2P	Alternating *k*-two-paths. Used as a “control” for alternating *k*-triangles
AS	Alternating *k*-stars. A positive parameter value indicates centralization based on high-degree nodes
AT	Alternating *k*-triangles. A positive parameter value indicates network closure (triangles)
Match *c*	Categorical matching on categorical attribute *c*. A positive parameter value indicates an edge preferentially forming between nodes with the same value of the categorical attribute (known as “homophily” in social network research)

**Table 3:** Parameters for directed networks

Effect	Description
Arc	Baseline density
Sink	A positive parameter value indicates a tendency for nodes with incoming but no outgoing arcs
Source	A positive parameter value indicates a tendency for nodes with outgoing but no incoming arcs
Reciprocity	A positive parameter value indicates a tendency for arcs to be reciprocated (a cycle of length 2)
AltInStars	Alternating *k*-in-stars. A positive parameter value indicates centralization based on high in-degree nodes
AltOutStars	Alternating *k*-out-stars. A positive parameter value indicates centralization based on high out-degree nodes
AltTwoPathsT	Multiple 2-paths. A positive parameter value indicates a tendency for directed paths of length 2. Used as a “control” for AltKTrianglesT, the parameter for triangles formed by closing these 2-paths
AltKTrianglesT	Path closure or transitive closure. A positive parameter value indicates a tendency for open directed two-paths to be closed transitively. This is an alternating statistic version of the “feed-forward loop” motif
AltKTrianglesC	Cyclic closure. A positive parameter value indicates a tendency for directed cycles of length 3 in the network, representing non-hierarchical network closure. An alternating statistic version of the “three-node feedback loop” motif
Sender *a*	Sender on binary attribute *a*. A positive parameter value indicates that nodes with the attribute are more likely to have an incident arc directed from them
Receiver *a*	Receiver on binary attribute *a*. A positive parameter value indicates that nodes with the attribute are more likely to have an incident arc directed to them
Interaction *a*	Interaction on binary attribute *a*. A positive parameter value indicates that two nodes which both have the attribute are more likely to have an arc directly connecting them
Matching *c*	Matching on categorical attribute *c*. A positive parameter value indicates that two nodes which have the same value of the attribute are more likely to have an arc directly connecting them
Loop	Self-edge. A positive parameter value indicates a tendency for self-edges (loops)

The “alternating” statistics (Lusher et al. 2013; Robins et al. 2007b; Snijders et al. 2006) such as alternating *k*-stars involve sums of counts of configurations with alternating signs and a decay factor $\lambda$, and, except where otherwise specified, we set $\lambda = 2$ in accordance with common ERGM modeling practice.

### ERGM parameter estimation

ERGM parameters for undirected networks were estimated using the EE algorithm (Byshkin et al. 2018) with the IFD sampler (Byshkin et al. 2016) implemented for undirected networks in the Estimnet software as described in Byshkin et al. (2018), with 20 estimations (run in parallel). ERGM parameters for directed networks were estimated using the simplified EE algorithm (Borisenko et al. 2019; Byshkin et al. 2018) with IFD sampler implemented for directed networks in the EstimNetDirected software (Stivala et al. 2020), with 64 estimations (run in parallel).

The Alon *E. coli* network does not contain any reciprocated arcs (directed loops of length two), and so estimation is made conditional on this by preventing the creation of reciprocated arcs in the MCMC procedure.

### Convergence and goodness-of-fit tests

Convergence was tested as described in Byshkin et al. (2018), Stivala et al. (2020), by requiring the absolute value of each parameter’s t-ratio to be no greater than 0.3, and by visual inspection of the parameter and statistic trace plots. For the directed networks estimated with EstimNetDirected, an additional heuristic convergence test was used, as described in Stivala et al. (2020). Observed graph statistics were plotted on the same plots as the distributions of those statistics in the networks simulated in the EE algorithm MCMC process, to check that they do not diverge. The statistics used are the same as those of the actual goodness-of-fit test described below, but note that this test is only for estimation convergence, not goodness-of-fit (Stivala et al. 2020).

For the directed networks estimated with EstimNetDirected, a simulation-based goodness of fit procedure was used, similar to that used in statnet (Hunter et al. 2008). A set of networks was simulated from the estimated model (using the SimulateERGM program in the EstimNetDirected software), and the distribution of certain graph statistics compared with those of the observed network by plotting the observed network values on the same plots as the distribution of simulated values. The statistics used were the in- and out-degree distributions, reciprocity, giant component size, mean local and global clustering coefficients, triad census, geodesic distance (shortest path length) distribution, and edge-wise and dyad-wise shared partners distributions.

## Results and discussion

Table 4 shows the basic structural model for the yeast PPI network (Model 1), a model with the alternating *k*-two-paths (A2P) parameter added (Model 2), as well as a model (Model 3) incorporating a parameter for the propensity of interactions to occur between proteins in the same functional category (class). Model 1 reproduces a model of this network in a previous work (Byshkin et al. 2018, Table S3); Models 2 and 3 are new.

**Table 4:** Parameter estimates with 95% confidence interval for the yeast PPI network, from the EE algorithm

Effect	Model 1	Model 2	Model 3
Edge	$\mathbf {\underset{(-7.806, -7.709)}{-7.758}}$	$\mathbf {\underset{(-10.685, -10.650)}{-10.667}}$	$\mathbf {\underset{(-9.302, -9.262)}{-9.282}}$
AS	${\underset{(-0.103, 0.007)}{-0.048}}$	$\mathbf {\underset{(1.013, 1.140)}{1.077}}$	$\mathbf {\underset{(0.550, 0.659)}{0.604}}$
A2P	–	$\mathbf {\underset{(-0.090, -0.084)}{-0.087}}$	$\mathbf {\underset{(-0.062, -0.056)}{-0.059}}$
AT	$\mathbf {\underset{(1.807, 1.907)}{1.857}}$	$\mathbf {\underset{(2.474, 2.548)}{2.511}}$	$\mathbf {\underset{(2.396, 2.467)}{2.432}}$
Match class	–	–	$\mathbf {\underset{(0.315, 0.402)}{0.358}}$

Parameter estimates that are statistically significant are shown in bold

Each of these model estimations took approximately 7 minutes total elapsed time on cluster nodes with Intel Xeon E5-2650 v3 2.30GHz processors using 20 parallel tasks.

We expect that proteins of the same functional category should preferentially interact with each other (von Mering et al. 2002), and this is confirmed by the significant positive parameter estimated for the “Match class” effect. The alternating *k*-triangle (AT) parameter is positive and significant in all models, showing an over-representation of triangles (which we might expect given the very high value of the clustering coefficient for this network, Table 1), even in models also including parameters for two-paths and preferential interaction of proteins in the same class.

Table 5 shows a basic structural model for the human PPI high confidence network (Model 1), and a model with a term to control for subellular location by categorical matching on the cellular component GO term (Model 2).

**Table 5:** Parameter estimates with 95% confidence interval for the human PPI (HIPPIE high confidence) network, from the EE algorithm

Effect	Model 1	Model 2
Edge	$\mathbf {\underset{(-12.550, -12.450)}{-12.500}}$	$\mathbf {\underset{(-12.498, -12.402)}{-12.450}}$
AS	$\mathbf {\underset{(1.222, 1.258)}{1.240}}$	$\mathbf {\underset{(1.208, 1.245)}{1.226}}$
A2P	$\mathbf {\underset{(-0.001, -0.001)}{-0.001}}$	$\mathbf {\underset{(-0.001, -0.001)}{-0.001}}$
AT	$\mathbf {\underset{(1.692, 1.711)}{1.701}}$	$\mathbf {\underset{(1.680, 1.699)}{1.690}}$
Match cellular component	–	$\mathbf {\underset{(0.431, 0.498)}{0.465}}$

Parameter estimates that are statistically significant are shown in bold

Estimation of Model 1 took approximately 64 minutes elapsed time, and Model 2 approximately 73 minutes, on cluster nodes with Intel Xeon E5-2650 v3 2.30GHz processors using 20 parallel tasks.

As discussed in the Introduction, we expect that interactions would be over-represented between proteins that share a subcellular location, and this is confirmed by a statistically significant positive parameter estimate for categorical matching on cellular component (Model 2 in Table 5). The alternating *k*-triangle (AT) parameter is positive and statistically significant in both models. This indicates an over-representation of triangles, even when controlling for subcellular location (Model 2).

We estimated four different models of the Alon *E. coli* regulatory network (Table 6). In Models 1 and 2, following Hummel et al. (2012), we modeled self-regulation by using a nodal covariate “self” which is true exactly when the node had a self-edge (loop) in the original network. These ERGM models are new, in that previous work with ERGMs on these networks either treated them as undirected (Hummel et al. 2012; Saul and Filkov 2007), thereby ignoring the inherently directed nature of such a regulatory network; or, in the case where the network was left as directed, included only Arc and alternating *k*-in-stars terms, as the estimation methods used at the time could not find converged models when other terms, such as triangles, were included (Begum et al. 2014).

**Table 6:** Parameter estimates with 95% confidence interval for the Alon *E. coli* regulatory network

Effect	Model 1	Model 2	Model 3	Model 4
Arc	$\mathbf {\underset{(-8.368, -8.047)}{-8.208}}$	$\mathbf {\underset{(-7.830, -7.509)}{-7.670}}$	$\mathbf {\underset{(-5.535, -5.149)}{-5.342}}$	$\mathbf {\underset{(-8.664, -8.337)}{-8.500}}$
Sink	$\underset{(-0.240, 6.830)}{3.295}$	$\mathbf {\underset{(0.306, 5.448)}{2.877}}$	$\underset{(-5.584, 4.330)}{-0.627}$	$\underset{(-1.512, 9.662)}{4.075}$
Source	$\underset{(-1.770, 4.247)}{1.238}$	$\underset{(-1.063, 4.156)}{1.546}$	$\underset{(-3.084, 5.195)}{1.056}$	$\underset{(-1.683, 6.551)}{2.434}$
AltInStars	$\mathbf {\underset{(0.943, 4.232)}{2.587}}$	$\mathbf {\underset{(0.555, 4.044)}{2.299}}$	$\underset{(-0.163, 3.392)}{1.614}$	$\mathbf {\underset{(0.630, 4.798)}{2.714}}$
AltOutStars	$\underset{(-2.363, 0.362)}{-1.001}$	$\underset{(-1.885, 0.189)}{-0.848}$	$\underset{(-3.987, 1.717)}{-1.135}$	$\underset{(-2.300, 1.266)}{-0.517}$
AltTwoPathsT	$\underset{(-0.638, 0.297)}{-0.170}$	$\underset{(-0.603, 0.272)}{-0.165}$	$\underset{(-1.376, 0.192)}{-0.592}$	$\underset{(-0.767, 0.267)}{-0.250}$
AltKTrianglesT	$\mathbf {\underset{(0.798, 4.972)}{2.885}}$	$\mathbf {\underset{(1.025, 4.636)}{2.830}}$	$\mathbf {\underset{(0.722, 5.555)}{3.139}}$	$\mathbf {\underset{(0.548, 5.604)}{3.076}}$
Matching self	–	$\underset{(-1.181, 0.280)}{-0.451}$	–	–
Loop	–	–	–	$\mathbf {\underset{(1.949, 14.256)}{8.103}}$

Parameter estimates that are statistically significant are shown in bold. In Models 3 and 4, self-edges (loops) are retained in the network and allowed in the model

Each of these model estimations took approximately three minutes total elapsed time on cluster nodes with Intel Xeon E5-2650 v3 2.30GHz processors using 64 parallel tasks.

**Fig. 5:**
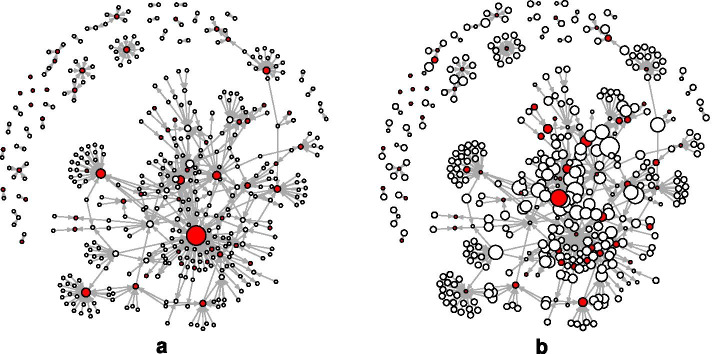
Alon *E. coli* regulatory network. (**a**) Node size is proportional to in-degree. (**b**) Node size is proportional to out-degree. Self-regulating operons are depicted as filled (red) circles. In (**a**) there appears to be a small set of high in-degree nodes and a much larger set of smaller in-degree nodes, while in (**b**) the out-degree of the nodes appears to be much more evenly distributed. The hypothesis we might make from (**a**), that there is centralization on in-degree, is confirmed by the ERGM results. This same model finds no support for the hypothesis we might make from (**b**), that there is a tendency against centralization on out-degree

In these models, the Sink and Source parameters are used to control, respectively, for the presence of genes that do not regulate any genes (have out-degree zero) and genes that are not regulated by any gene (have in-degree zero). The alternating *k*-in-stars (AltInStars) parameter is positive and significant in all models except Model 3, indicating significant skewness of the in-degree distribution, that is, the presence of “hubs” with higher in-degree than other nodes. There is no significant effect for (or against) such skewness of the out-degree distribution (see Figs. 3 and 5).

The only other parameter that is consistently significant (and positive) is path closure (AltKTrianglesT), which we can interpret as a significant tendency for the “feed-forward loop” to be over-represented, consistent with the results in Milo et al. (2002).

**Fig. 6:**
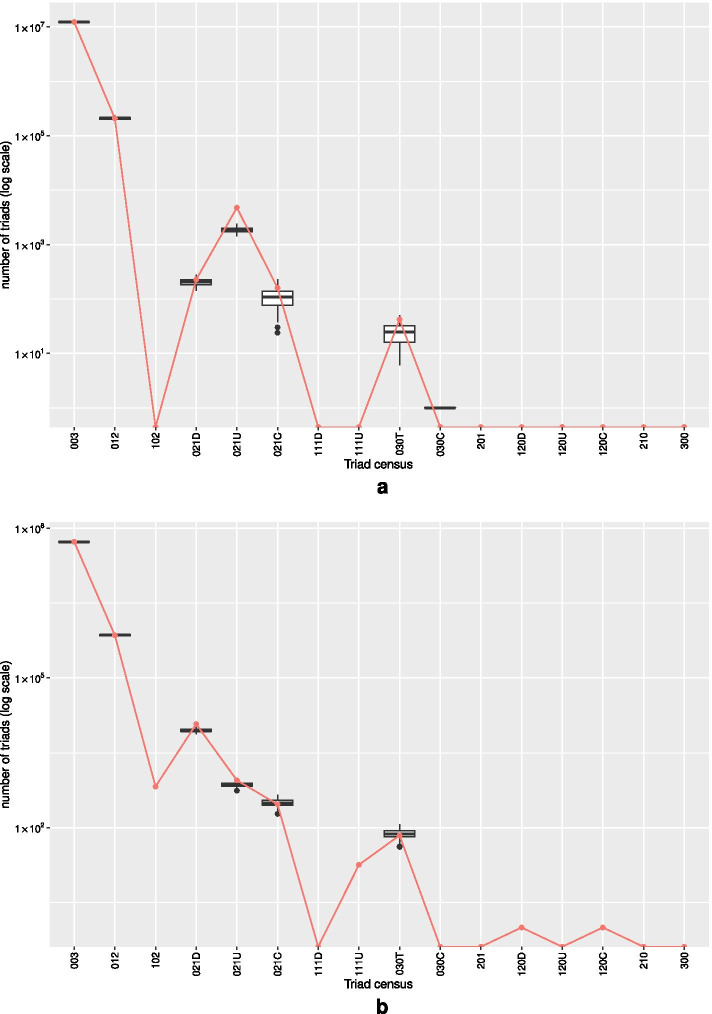
Goodness-of-fit plots for the triad census of (**a**) the Alon *E. coli* regulatory network, Model 1 (Table 6), and (**b**) the Alon yeast regulatory network, Model 1 (Table 7). The observed triad counts are plotted in red with the triad counts of 100 simulated networks plotted as black boxplots. Because the triad counts (*y*-axis) are on a log scale, values of zero are omitted (observed zero counts shown as a red point on the bottom of the graph). In (**a**), for triad census class 030C (cyclic triad), the “box plot” consisting of a single median line for the simulated count represents a single (out of 100 simulations) occurrence of a nonzero count (of 1) for 030C

A goodness-of-fit plot for Model 1 (Table 6) is shown in Additional file 1: Fig. S1a, showing a good fit for the model. A goodness-of-fit plot for the triad census (Fig. 6a) shows that the model reproduces the triad census well, and specifically triad 030T, the transitive triad (three node feed-forward loop), giving additional confidence that the positive and statistically significant AltKTrianglesT parameter is evidence for over-representation of this motif, given the other parameters in the model.

Note that this *E. coli* regulatory network does not contain any instances of the three-cycle, or “three-node feedback loop” (Milo et al. 2002). Indeed the Alon *E. coli* network does not contain any loops greater than size one (Shen-Orr et al. 2002), and so the cyclic closure parameter (AltKTrianglesC) is not included in the models.

In Models 3 and 4 (Table 6), unlike the other models, self-edges (loops) are retained in the network, and self-edges are allowed in the modeling process, allowing the formation of loops to be modeled jointly with the other structural features in the model.^1^ In Model 4, the new parameter “Loop” is introduced, for which the corresponding statistic is the count of self-edges in the network. This parameter is statistically significant and positive, indicating that self-edges are over-represented, given the other effects included in the model. Goodness-of-fit plots for Models 3 and 4 (Table 6) are shown in Additional file 1: Fig. S4, showing that when the Loop parameter is not included in the model (Model 3 in Table 6), there is a poor fit for the number of loops (Additional file 1: Fig. S4a). However, when the Loop parameter is included (Model 4 in Table 6), there is a good fit for the number of loops (Additional file 1: Fig. S4b).

We found that it is also possible to estimate similar models of this relatively small network using the most recent version of the statnet ergm package (Handcock et al. 2021; Krivitsky et al. 2021), with the “stepping” algorithm (Hummel et al. 2012). These models are shown in Additional file 1: Table S1, and the goodness-of-fit plots in Additional file 1: Figs. S6, S7. The results are consistent with those in Table 6. Specifically, there is a significant positive estimate for geometrically weighted edge-wise shared partners (GWESP, equivalent to AltKTrianglesT), and a significant negative estimate for geometrically weighted in-degree, indicating centralization in the in-degree distribution.^2^ The statnet model finds a significant tendency against centralization on out-degree, while the models in Table 6 did not have a significant estimate for the corresponding parameter (AltOutStars). Similarly the statnet model (Model 2 in Additional file 1: Table S1) finds a significant negative parameter estimate for Matching on the “self-regulating” attribute, while no significant effect is found in Model 2 in Table 6. The statnet ergm package does not allow for the modeling of self-edges, however (Hummel et al. 2012).

Table 7 shows ERGM parameter estimates for the Alon yeast regulatory network. Each of these model estimations took approximately three minutes total elapsed time on cluster nodes with Intel Xeon E5-2650 v3 2.30GHz processors using 64 parallel tasks. These ERGM models are also new; previously published ERGMs for similar networks having treated them as undirected (Saul and Filkov 2007).

**Table 7:** Parameter estimates with 95% confidence intervals for the Alon yeast regulatory network

Effect	Model 1	Model 2	Model 3
Arc	$\mathbf {\underset{(-7.665, -7.313)}{-7.489}}$	$\mathbf {\underset{(-7.662, -7.318)}{-7.490}}$	$\mathbf {\underset{(-7.667, -7.313)}{-7.490}}$
Reciprocity	—	—	$\underset{(-15.535, 3.307)}{-6.114}$
AltInStars	$\underset{(-1.504, 0.577)}{-0.463}$	$\underset{(-1.451, 0.581)}{-0.435}$	$\underset{(-1.472, 0.568)}{-0.452}$
AltOutStars ($\lambda = 4.5$)	$\mathbf {\underset{(0.756, 1.261)}{1.008}}$	$\mathbf {\underset{(0.757, 1.256)}{1.006}}$	$\mathbf {\underset{(0.752, 1.264)}{1.008}}$
AltTwoPathsT ($\lambda = 3$)	$\underset{(-0.739, 0.076)}{-0.332}$	$\underset{(-0.670, 0.074)}{-0.298}$	$\underset{(-0.684, 0.054)}{-0.315}$
AltKTrianglesT ($\lambda = 3$)	$\mathbf {\underset{(0.484, 4.111)}{2.297}}$	$\mathbf {\underset{(0.043, 3.640)}{1.842}}$	$\mathbf {\underset{(0.479, 3.763)}{2.121}}$

Parameter estimates that are statistically significant are shown in bold. In Model 1 only, estimation is conditional on no reciprocated arcs, even though there is a single reciprocated arc in the data. Model 3 is included for illustration, even though it shows poor convergence with respect to the Reciprocity parameter (t-ratio magnitude is greater than 0.3)

In Model 1 (Table 7), estimation is conditional on no reciprocated arcs, just as was done for the *E. coli* regulatory network. However in this yeast regulatory network, there is actually a single reciprocated arc (two-cycle) in the data, and hence the fit of the model on statistics involving reciprocated arcs is poor. This is apparent, for example, in the poor fit for triad census class 102 (triad with only a mutual arc) in Fig. 6b, or for the reciprocity statistic in the goodness-of-fit plot (Additional file 1: Fig. S1b). The fit for other statistics, and in particular the degree and shared partner distributions, is acceptable (with the exception of poor fit on the giant component size). Importantly, the fit on the triad census class 030T (transitive triad) is good (Fig. 6b).

In order to better model reciprocity, a model (Model 2 in Table 7) was estimated without being conditional on there being no reciprocated arcs, but without a reciprocity term in the model. This model also has adequate goodness-of-fit, but this time including good fit on the reciprocity statistic (Additional file 1: Fig. S2a). It does, however, for some triads involving reciprocated arcs (120U for example), generate significantly more such triads than are observed in the data (Additional file 1: Fig. S2b). Therefore, a third model (Model 3 in Table 7) was estimated, including the Reciprocity parameter. However, probably due to the fact that the data contains only a single reciprocated arc, this model has a very large estimated standard error for the Reciprocity parameter. Further, it exhibits poor convergence with respect to the Reciprocity statistic, with a t-ratio greater than the maximum value of 0.3 we consider acceptable, since the data contains exactly one reciprocated arc, yet the model most frequently generates networks with none.

Model 1 and Model 2, therefore, are preferable. Nevertheless, in all three models, the sign and significance of estimated parameters (except Reciprocity) are the same. There is a positive and significant parameter for alternating *k*-out-stars (AltOutStars), indicating the presence of “hubs” with higher out-degree than other nodes. This is as we might expect from Fig. 3 and previous research (Balaji et al. 2006; Guelzim et al. 2002; Monteiro et al. 2020; Ouma et al. 2018), and contrasts with the *E. coli* regulatory network, which has in-degree hubs but not out-degree hubs.

Also in all three models, there is a positive and significant parameter estimate for transitive closure (AltKTrianglesT). Given this estimate, and the good fit for the transitive closure motif 030T (Fig. 6b) we can again interpret this as a significant over-representation of this motif (“feed-forward loop”), consistent with the results of Milo et al. (2002).

In all three models in Table 7, the decay parameter $\lambda$ for the “alternating” statistics has been set to a value other than the default $\lambda =2$ for alternating *k*-out-stars (AltOutStars), multiple two-paths (AltTwoPathsT), and transitive closure (AltKTrianglesT). This is because models initially estimated with the default $\lambda =2$ value (Additional file 1: Table S2) showed poor goodness-of-fit on the out-degree distribution (Additional file 1: Fig. S3a) and triad census class 030T (Additional file 1: Fig. S3b). Therefore, new models were estimated with a higher value of $\lambda$ for the alternating *k*-out-star parameter to assist with modeling the highly skewed out-degree distribution (Koskinen and Daraganova 2013), and also a higher value of $\lambda$ for AltTwoPathsT and AltKTrianglesT (the same value of $\lambda$ for both) to aid model convergence and fit for transitivity (Snijders et al. 2006).

As with the *E. coli* network, we also estimated a model of the yeast regulatory network, in which self-edges are retained, and allowing self-edges (loops) in the model. This network (even leaving aside the presence of self-edges) is, however, not identical to the network used for the models shown in Table 7, having two additional nodes. Its graph summary statistics, are, however the same (to the precision shown) as those of the version shown in Table 1, other than it having 690 rather than 688 nodes. Since the network modeled is a slightly different network than that used for the models shown in Table 7, these models are presented separately, in Additional file 1: Table S3. The results are consistent with those in Table 7, with statistically significant positive parameter estimates for AltOutStars and AltKTrianglesT. The estimate for the Loop parameter is not statistically significant, however. Goodness-of-fit plots for the models in Additional file 1: Table S3 are shown in Additional file 1: Fig. S5. These figures show that the model which allows self-edges, but does not include the Loop parameter (Model 1 in Additional file 1: Table S3) does not fit the number of loops well, while the model that includes the Loop parameter (Model 2 in Additional file 1: Table S3) does fit the number of loops well.

The cyclic triangle structure has been suggested as an “anti-motif” (i.e. occurs less frequently than expected), but in some cases its apparent under-representation has been shown to be an expected consequence of other topological properties of biological networks (Konagurthu and Lesk 2008a). This closed-loop structure, also known as a “multicomponent loop”, can provide feedback control and potentially produce systems that can switch between two states (Ferrell 2002; Lee et al. 2002). In the examples used here, there were so few (or no) occurrences of this motif, that models including the corresponding parameter (in the form of the AltKTrianglesC parameter) would not converge. Yet the networks simulated from these models also contain no (or very few) occurrences of this candidate anti-motif. This is consistent with the lack of cyclic triangles not being due to cyclic triangles being an anti-motif as such, but rather as a consequence of the other topological features of the network, and specifically in these examples, the features described by the parameters included in the models. This is not a new finding, it having previously been noted that the lack of three-node feedback loops in the *E. coli* regulatory network (Lee et al. 2002; Shen-Orr et al. 2002) is reproduced in randomized networks (Shen-Orr et al. 2002).

The biological significance of the feed-forward loop (transitive triangle) is suggested to be that, by providing two pathways to affect the output, one direct, and one through an intermediate link, it can act as a logical “AND” gate, and filter out transient activation signals (Alon 2007; Lesk and Konagurthu 2021; Mangan and Alon 2003; Shen-Orr et al. 2002). Whether or not this is indeed the biological function of the feed-forward loop (Mazurie et al. 2005), this motif is found to be significantly over-represented in the transcriptional regulatory networks of several organisms (Alon 2007), including the yeast and *E. coli* networks studied here, and the feed-forward loop has been described as “highly favored during the evolution of transcriptional regulatory networks in yeast” (Lee et al. 2002, p. 801).

More recently, there has been interest in trying to understand the function of motifs by examining higher levels of structure. Gorochowski et al. (2018) examine the clustering of motifs, including the feed-forward loop, and find that a measure of motif clustering diversity can predict functionally important nodes in the *E. coli* metabolic network. Lesk and Konagurthu (2021) describes how the local structure of the yeast regulatory network is reconfigured in different physiological states.

So far we have only discussed results for three-node motifs, such as the feed-forward loop. We can test for the over-representation of other motifs, without including parameters for them in the model, by using the ERGM as the null model against which to compare the count of the motif in the observed network. This was the technique used by Felmlee et al. (2021), for example.

**Fig. 7:**
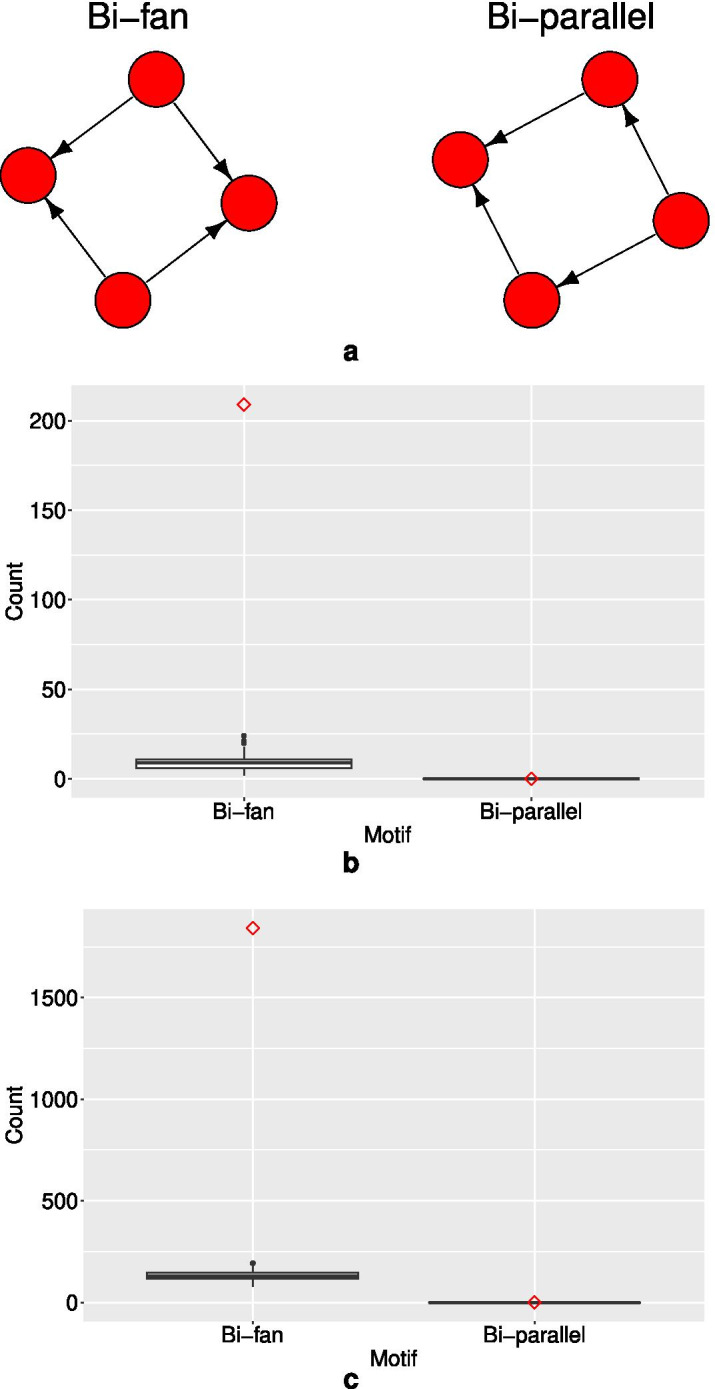
(**a**) The bi-fan and bi-parallel four-node motifs. Goodness-of-fit plots for these motifs for (**b**) the Alon *E. coli* regulatory network Model 1 (Table 6), and (**c**) the Alon yeast regulatory network Model 1 (Table 7). The observed network statistics are plotted as a red diamond, with the statistics of 100 simulated networks plotted as black boxplots

Figure 7 shows the bi-fan and bi-parallel motifs, as defined by Milo et al. (2002), their counts in the *E. coli* and yeast regulatory networks, and their distribution in ERGM models of these networks. The motifs were counted with the NetMODE software (Li et al. 2012). Note that NetMODE was used only to count the motifs, not to simulate any networks, which are simulated from the ERGM models as described in the Methods section.

The bi-parallel motif occurs in neither of the observed networks, and nor does it occur in any of the networks simulated from the corresponding ERGMs. The bi-fan motif, however, clearly occurs far more frequently in both observed networks than it does in the corresponding simulated networks. Note that these networks are simulated from ERGMs that model not just degree distribution, but also the distribution of two-paths and transitive triangles. Therefore, this shows that the bi-fan motif appears to be over-represented in the observed networks, even given the over-representation of transitivity captured in the models, which also reasonably reproduce the triad census, geodesic distance distribution, and dyad-wise and edge-wise shared partner distributions. These results are consistent with the results of Milo et al. (2002), where only degree-preserving randomization was used.

## Limitations

Finding a converged ERGM for a network is not always possible in practice. In particular, models which include Markov dependency assumption parameters such as triangles, corresponding directly to three-node motif candidates such as three-node feed-forward-loops (transitive triangles) and three-cycles, for example, usually do not converge. For this reason it is normal practice in ERGM modeling to use geometrically weighted or “alternating” configurations to solve this problem (Hunter et al. 2012; Robins et al. 2007b; Snijders et al. 2006), as we did in this work. However this means we are not answering precisely the same question as when we ask directly if a motif is over-represented or not. This is because ERGM is a model for tie (edge or arc) formation, not for motif formation: if we consider ERGM as a type of logistic regression, the outcome variable is the presence or absence of a network tie. The predictor variables are not independent of each other, but form a nested hierarchy of configurations: triangles are formed by “closing” a two-path with an additional edge, for example. So a positive estimate of the alternating *k*-triangle parameter does not directly mean that the transitive triangle (three node feed-forward loop) motif is over-represented, but rather that there is tendency (that is, it is more probable than chance given the other parameters in the model) for three nodes forming a directed two-path to be closed in a transitive triangle. This makes sense in the social network origins of the model: it might be assumed to be the result in the observed network of the tendency of a person’s friends to also be friends with each other, for example. In the context of biological networks, it might be interpreted as a sign of evolutionary events, however this interpretation is very much open to question, as briefly discussed in the Introduction.

Even when the “alternating” configurations are used, it can be difficult or impossible to find a converged and well-fitting ERGM for a given network. For example, we were unable to fit an ERGM with triangular configurations (using either statnet or EstimNetDirected) to an example of a neural network, the whole-animal chemical connectome (a directed network with 579 nodes and 5246 arcs) of the male *C. elegans* worm (Cook et al. 2019).

Hence in order to directly test motif significance, without having to fit a parameterized model such as ERGM, new methods, such as the “anchored motif” proposed by Fodor et al. (2020) are still required.

In some of the models presented here, we used values other than the usual default value $\lambda =2$ for the decay parameter $\lambda$ of the “alternating” statistics. We had to manually estimate appropriate values of $\lambda$ based on trial and error, guided by knowledge of the observed network, convergence and goodness-of-fit of the models (or lack thereof), and the definitions of the relevant statistics (Koskinen and Daraganova 2013; Snijders et al. 2006). It is possible to instead estimate $\lambda$ (or an equivalent parameter) directly from the data, as part of the model, using a “curved ERGM” (Hunter 2007; Hunter and Handcock 2006), and this is implemented in the statnet R package (Handcock et al. 2008, 2016, 2021; Hunter et al. 2008; Krivitsky et al. 2021; Morris et al. 2008). However it is not currently possible to estimate curved ERGMs using the EstimNetDirected software (Stivala et al. 2020), and this is an area requiring further work. In the absence of such a principled way of estimating the decay parameters, an alternative to the heuristic (trial and error) approach used here is to estimate many models with systematically varying values of the $\lambda$ decay parameter for each relevant “alternating” model parameter, and use a grid search to find the model with best fit.^3^ We applied this method to the Alon yeast regulatory network model (Additional file 1: Table S2), using the Mahalanobis distance between a vector of some of the observed network summary statistics used for goodness-of-fit (degree distributions, reciprocity, giant component size, global and average local clustering coefficient), and the corresponding vectors for networks simulated from the model, as the value to minimize. We used a two-dimensional grid, varying the $\lambda$ value for AltOutStars as one dimension, and the value of $\lambda$ for both AltTwoPaths and AltKTriangles (these values should be the same, as described in Snijders et al. (2006)) as the other dimension. With both values varying from 1.5 to 5.0 in steps of 0.5, we found the minimum Mahalanobis distance was at $\lambda = 4.5$ for the AltOutStars parameter, and $\lambda = 1.5$ for the AltTwoPathsT and AltKTrianglesT parameters. The parameters estimated for this model are not substantively different from those in Table 7. The values of $\lambda$ that we determined heuristically (Table 7) were at rank 15 (of 64) using this criterion. The model with the default $\lambda = 2.0$ for all alternating statistic parameters, with subjectively poor goodness-of-fit on the out-degree distribution, is at rank 48 (of 64).

As previously mentioned, the configurations available in an ERGM are determined by the dependence assumptions: although there is a lot of flexibility available in ERGM configurations, we cannot simply add arbitrary configurations without regard for the underlying dependency assumption (Koskinen 2020). The least restrictive assumption used in practice is the “social circuit” dependency assumption (Lusher et al. 2013; Robins et al. 2007b, 2009; Snijders et al. 2006) used in this work, which allows the use of the “alternating” configurations.

We also note that some recent work suggests that complex network structure, including heavy-tailed degree distributions, closure (clustering), large connected components, and short path lengths can arise simply from thresholding normally distributed data to generate the binary network (Cantwell et al. 2020). Hence inferences from ERGM modeling about network structure, just as with other techniques such as comparison to ensembles of random graphs, could be consequences of the way the binary network was constructed.

Valued ERGMs (Desmarais and Cranmer 2012; Krivitsky 2012) may be used to avoid this problem by removing the need to construct a binary network at all, and working directly with the network with valued edges. Parameter estimation for these models is even more computationally intensive than for binary networks, and hence is so far impractical to use for networks of the size considered here. Using new estimation techniques to improve the scalability of parameter estimation for valued ERGMs is another area requiring further research.

For the relatively small (on the order of one thousand nodes or fewer) directed networks considered here, it is possible to do simulation-based goodness-of-fit tests. However, it is possible to estimate ERGM parameters for far larger (over one million nodes) networks using the EstimNetDirected software, but it is not practical to simulate such large networks from the model, and this is an area requiring further work (Stivala et al. 2020).

One further limitation to consider is the execution time of the ERGM technique. As discussed in the introductory sections, ERGM parameter estimation is a computationally difficult problem. Although recent advances allow the estimation in minutes of models that would have taken hours, or been infeasible to estimate, with earlier methods, it is still much more computationally difficult to do this than it is to run conventional motif finding methods. The networks used here took between three and 73 minutes to estimate, using multiple (up to 64) processor cores in parallel. However motif finding with MFinder (Kashtan et al. 2004) in these networks takes only seconds, and with the faster NetMODE method (Li et al. 2012), even less time, using only a single processor core.

## Conclusion

We have re-examined the use of exponential random graph models for analyzing biological networks, an application first introduced in the bioinformatics literature by Saul and Filkov (2007). Advances in ERGM estimation methods since then have allowed more sophisticated models to be estimated for more and larger networks than was possible at the time, and they are now a more practical technique for making inferences about structural hypotheses in biological networks, potentially solving some of the problems inherent in conventional methods for testing motif over-representation. By using an ERGM, all configurations in the model are tested simultaneously, each conditional on all the others, rather than having to test one at a time with the other configurations fixed in a (more or less sophisticated, the choice of which is critical to the results) null model.

The ERGM models of the Alon *E. coli* network presented here are the first to retain the directed nature of the network and also include terms for triangular structures. They confirm the result of Milo et al. (2002) that path closure (feed-forward loop) is over-represented, even when we include other, related, parameters in the model.

We also presented the first ERGM models of a yeast regulatory network retaining its inherently directed nature (rather than treating it as undirected). We find statistically significant over-representation of the transitive closure motif, just as Milo et al. (2002) did in the same yeast regulatory network, using a simple randomization test.

The lack of the cyclic triangle (feedback loop) structure in the data, however, is reproduced by models that do not contain any parameter corresponding to this structure. This suggests that this structure is not an “anti-motif”, but rather that its lack is a consequence of the structural features of the networks, specifically degree distributions, two-paths, and transitive closure, that are included in the models.

## Supplementary Information

**Additional file 1: Supplementary tables and figures. **Additional models and goodness-of-fit plots.

## Data Availability

Source code, configuration files, and datasets are available from https://sites.google.com/site/alexdstivala/home/ergm_bionetworks.

## Abbreviations

CDF: Cumulative distribution function
CUG: Conditional uniform graph
DMN: Default mode network
EE: Equilibrium expectation
EEG: Electroencephalography
ERGM: Exponential random graph model
GO: Gene ontology
HIPPIE: Human integrated protein–protein interaction reference
IFD: Improved fixed density
MAN: Mutual, asymmetric, null
MCMC: Markov chain Monte Carlo
PANTHER: Protein analysis through evolutionary relationships
PPI: Protein–protein interaction
SBM: Stochastic block model

## Footnotes

1 Modeling self-edges in this way was suggested by an anonynous reviewer, on the grounds that a node with a self-edge is a (very simple) motif.

2 Note that a *negative* estimate of the geometrically weighted degree parameter in statnet has the same interpretation as a *positive* estimate for the alternating *k*-stars parameter as used here. This frequently leads to confusion (Levy 2016; Levy et al. 2016).

3 This strategy was suggested by an anonymous reviewer.
