# Supplementary material for: Testing biological network motif significance with exponential random graph models
Source: Appl Netw Sci. 2021 Nov 22;6(1):91. doi: 10.1007/s41109-021-00434-y (PMC8608783; doi:10.1007/s41109-021-00434-y)
Supplement: Supplementary file 1 — Additional file 1: Supplementary tables and figures. Additional models and goodness-of-fit plots. [file 41109_2021_434_MOESM1_ESM.pdf]

Testing biological network motif significance with exponential random graph models
Additional file 1

Alex Stivala[1], Alessandro Lomi[1,2]
[1]Università della Svizzera italiana, Via Giuseppe Buffi 13, 6900 Lugano, Switzerland
[2]The University of Exeter Business School, Rennes Drive, Exteter EX4 4PU,United Kingdom
alexander.stivala@usi.ch
alessandro.lomi@usi.ch

Supplementary tables

Table S1: Parameter estimates for the Alon *E. coli* regulatory network, estimated using the "stepping" algorithm (Hummel et al, 2012) in the statnet ergm package (Handcock et al, 2008; Morris et al, 2008; Hunter et al, 2008; Handcock et al, 2016, 2021; Krivitsky et al, 2021). The decay parameter α for geometrically weighted dyad-wise shared partners (GWDSP) and geometrically weighted edge-wise shared partners (GWESP) OTP ("outgoing two path", that is, transitive shared partner) is set to $\log(2.0)$, equivalent to the default value $\lambda = 2.0$ for AltTwoPaths and AltKTrianglesT in the EstimNetDirected software.

Effect	Model 1	Model 2
Edges	-3.113 $(0.077)^{***}$	-3.025 $(0.066)^{***}$
GW in-degree ($\alpha = 2$)	-3.954 $(0.124)^{***}$	-3.818 $(0.140)^{***}$
GW out-degree ($\alpha = 0$)	1.588 $(0.184)^{***}$	1.578 $(0.190)^{***}$
GWDSP OTP ($\alpha = 0.693$)	-0.493 $(0.077)^{***}$	-0.502 $(0.080)^{***}$
GWESP OTP ($\alpha = 0.693$)	2.378 $(0.201)^{***}$	2.382 $(0.193)^{***}$
Nodematch self		-0.271 $(0.086)^{**}$
AIC	5445.00	5426.00
BIC	5495.00	5487.00

*** $p < 0.001$; ** $p < 0.01$; * $p < 0.05$; $^{\cdot}p < 0.1$.

Table S2: Parameter estimates for the Alon yeast regulatory network, with the default $\lambda = 2$ for the "alternating" parameters.

Effect	Model 1	Model 2	Model 3
Arc	$\mathbf{-10.578}$ $(\mathbf{-10.820},\mathbf{-10.335})$	$\mathbf{-10.080}$ $(\mathbf{-10.331},\mathbf{-9.828})$	$\mathbf{-10.093}$ $(\mathbf{-10.342},\mathbf{-9.844})$
Sink	3.124 (−3.407,9.655)	3.039 (−3.644,9.721)	3.003 (−3.637,9.642)
Source	2.903 (−4.488,10.295)	1.601 (−6.715,9.917)	1.949 (−6.490,10.389)
Reciprocity	—	—	−10.800 (−26.307,4.707)
AltInStars	0.347 (−2.723,3.416)	0.427 (−2.804,3.658)	0.336 (−2.818,3.490)
AltOutStars	2.860 (−0.278,5.998)	2.585 (−0.515,5.685)	2.637 (−0.537,5.811)
AltTwoPathsT	—	−0.110 (−0.581,0.360)	−0.149 (−0.607,0.310)
AltKTrianglesT	$\mathbf{1.227}$ $(\mathbf{0.315},\mathbf{2.139})$	1.706 (−0.773,4.185)	$\mathbf{2.390}$ $(\mathbf{0.011},\mathbf{4.770})$

Parameter estimates that are statistically significant at the 95% level are shown in bold. Model 3 is included for illustration, even though it shows poor convergence with respect to the Reciprocity parameter (t-ratio magnitude is greater than 0.3)

Table S3: Parameter estimates for the Alon yeast regulatory network, with self-edges included. The decay parameters λ are set to the best value found by grid search in the network without self-edges, as described in the main text.

Effect	Model 1	Model 2
Arc	$\mathbf{-7.430}$ $(\mathbf{-7.606},\mathbf{-7.254})$	$\mathbf{-7.521}$ $(\mathbf{-7.702},\mathbf{-7.340})$
Loop	—	1.720 (−3.650,7.091)
AltInStars	−0.466 (−1.498,0.566)	−0.414 (−1.414,0.585)
AltOutStars ($\lambda = 4.5$)	$\mathbf{1.000}$ $(\mathbf{0.757},\mathbf{1.243})$	$\mathbf{1.005}$ $(\mathbf{0.757},\mathbf{1.252})$
AltTwoPathsT ($\lambda = 1.5$)	−0.304 (−0.675,0.068)	−0.294 (−0.672,0.084)
AltKTrianglesT ($\lambda = 1.5$)	$\mathbf{1.884}$ $(\mathbf{0.059},\mathbf{3.709})$	$\mathbf{1.868}$ $(\mathbf{0.031},\mathbf{3.704})$

Parameter estimates that are statistically significant at the 95% level are shown in bold.

Supplementary figures

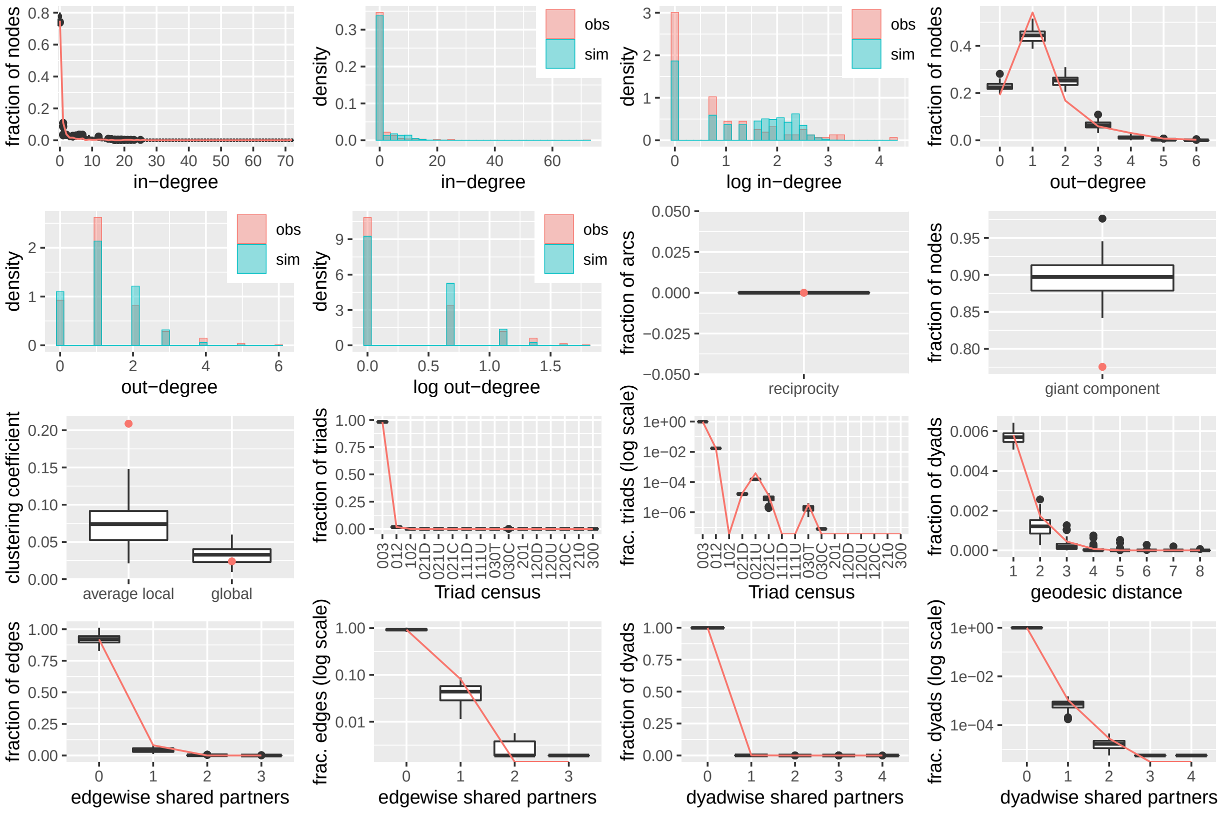

a

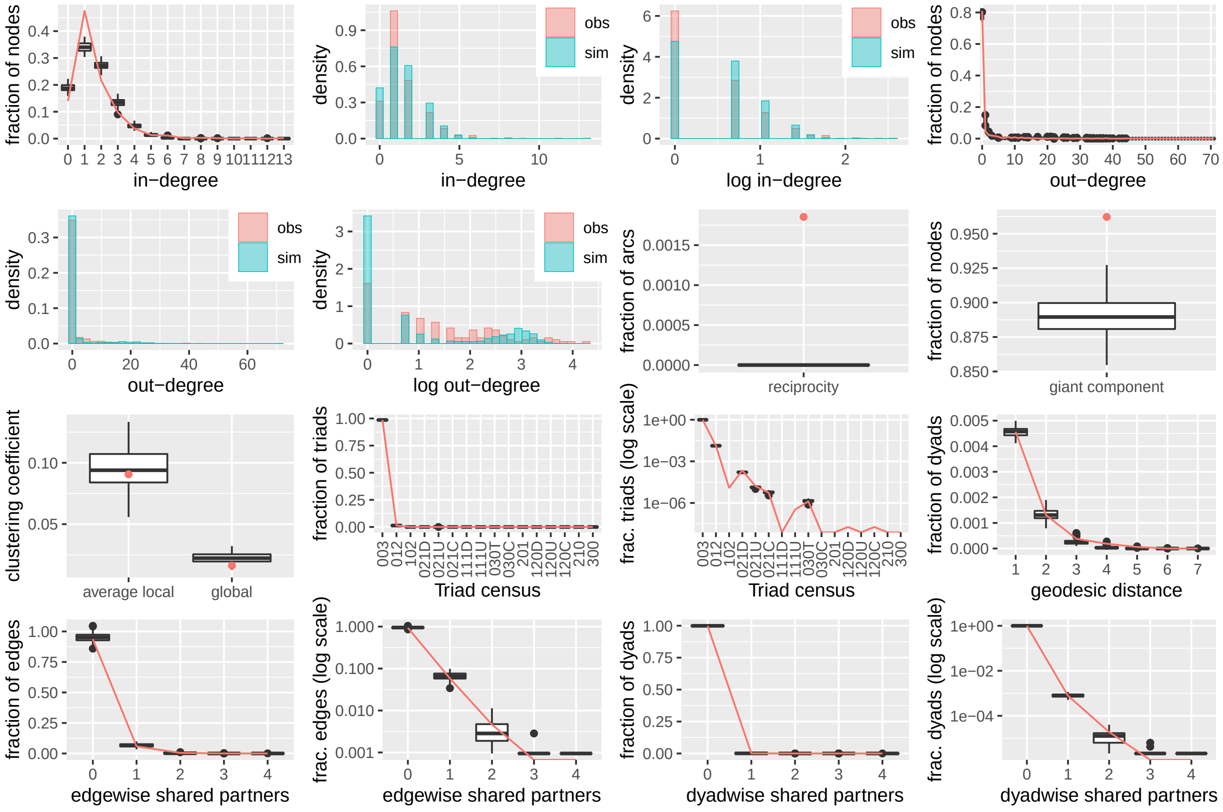

b

Figure S1: Goodness-of-fit plots for (**a**) the Alon *E. coli* regulatory network Model 1 (Table 6 in the main text), and (**b**) the Alon yeast regulatory network Model 1 (Table 7 in the main text). The observed network statistics are plotted in red with the statistics of 100 simulated networks plotted as black boxplots, and blue on the histograms.

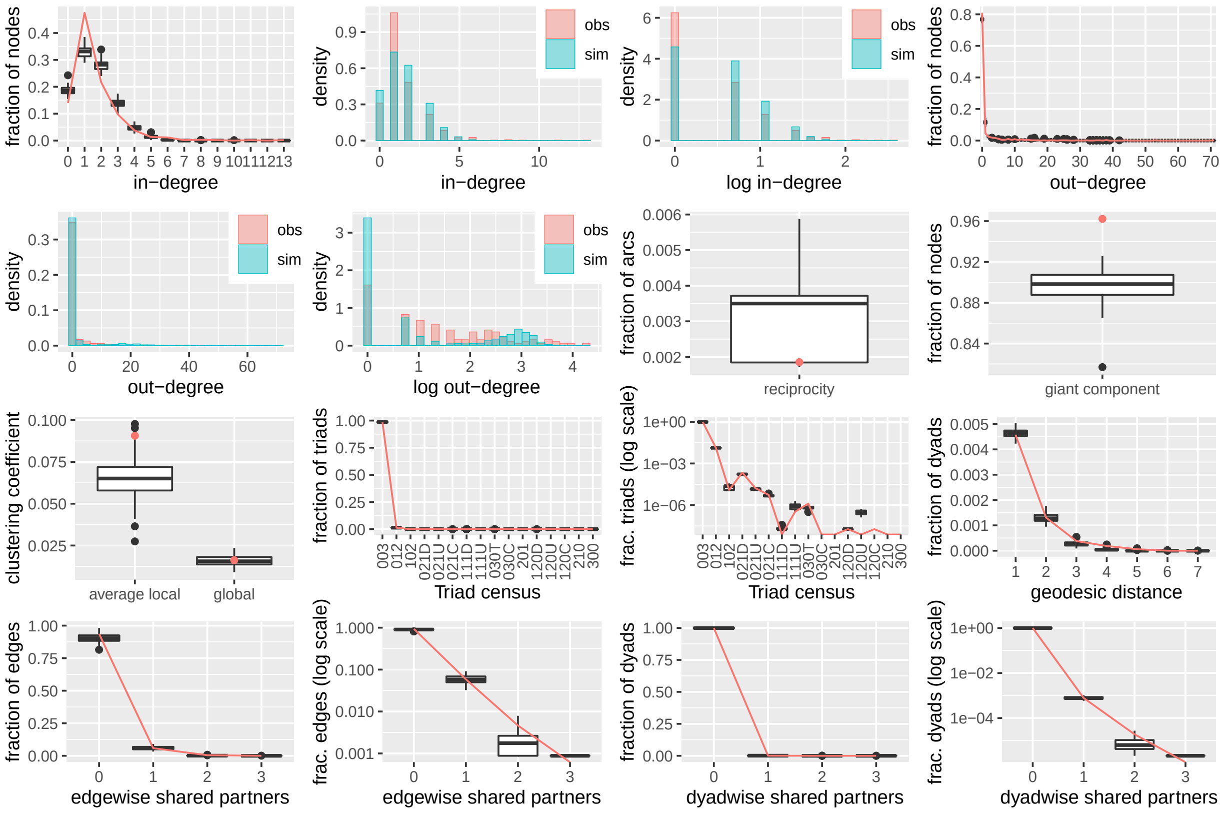

a

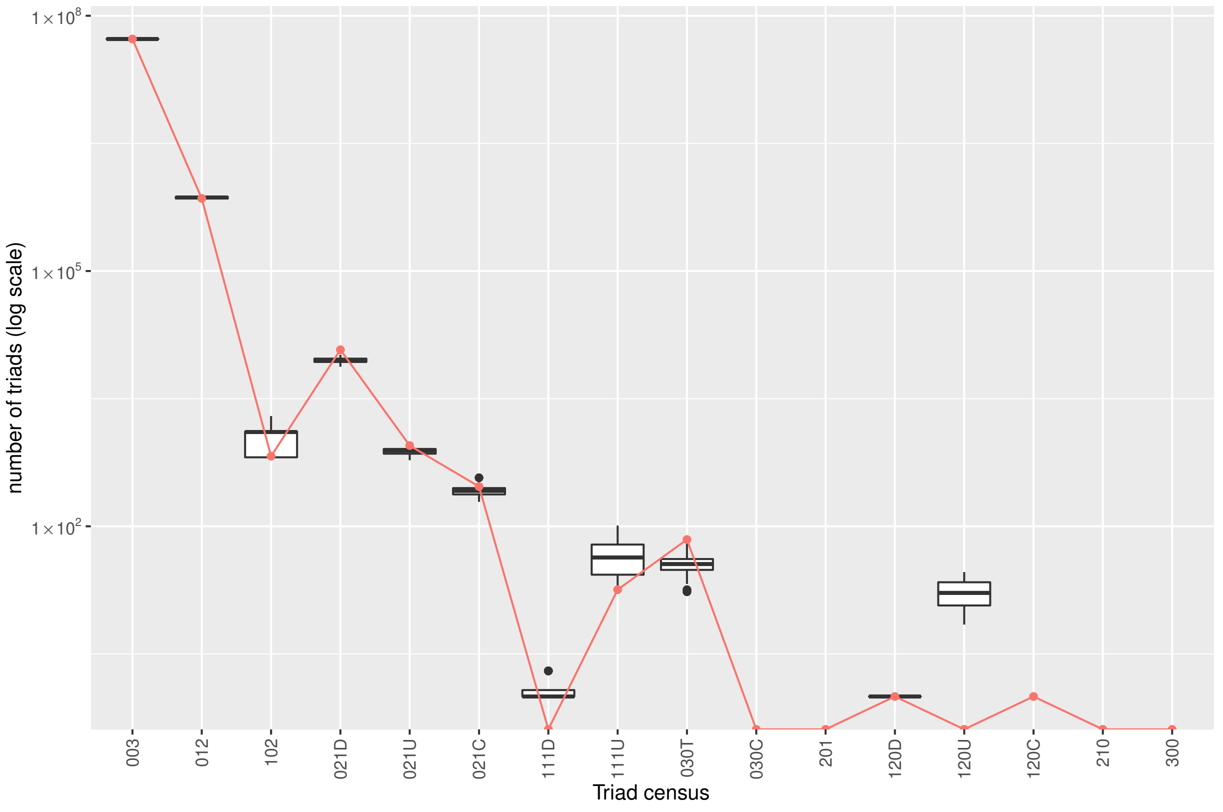

b

Figure S2: (**a**) Goodness-of-fit plots, and (**b**) triad census goodness-of-fit plot, for the Alon yeast regulatory network Model 2 (Table 7 in the main text). The observed network statistics are plotted in red with the statistics of 100 simulated networks plotted as black boxplots, and blue on the histograms.

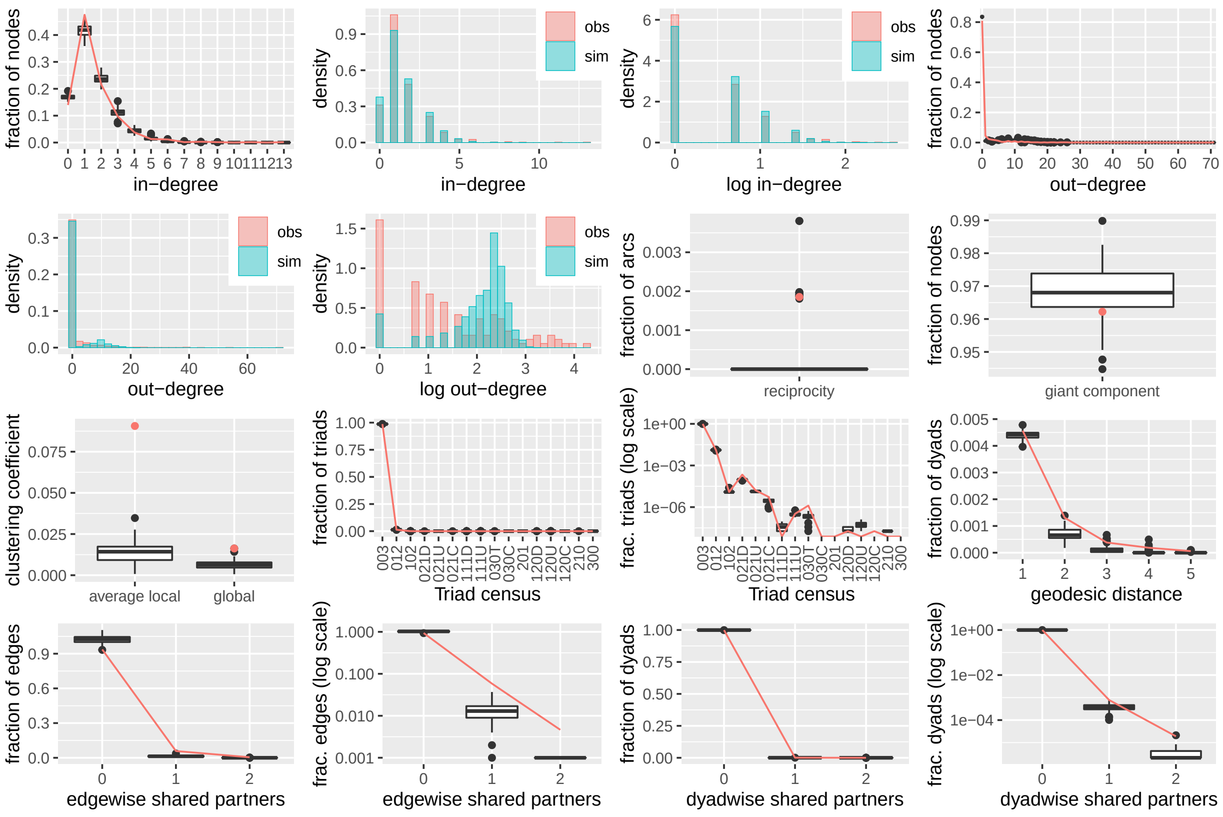

a

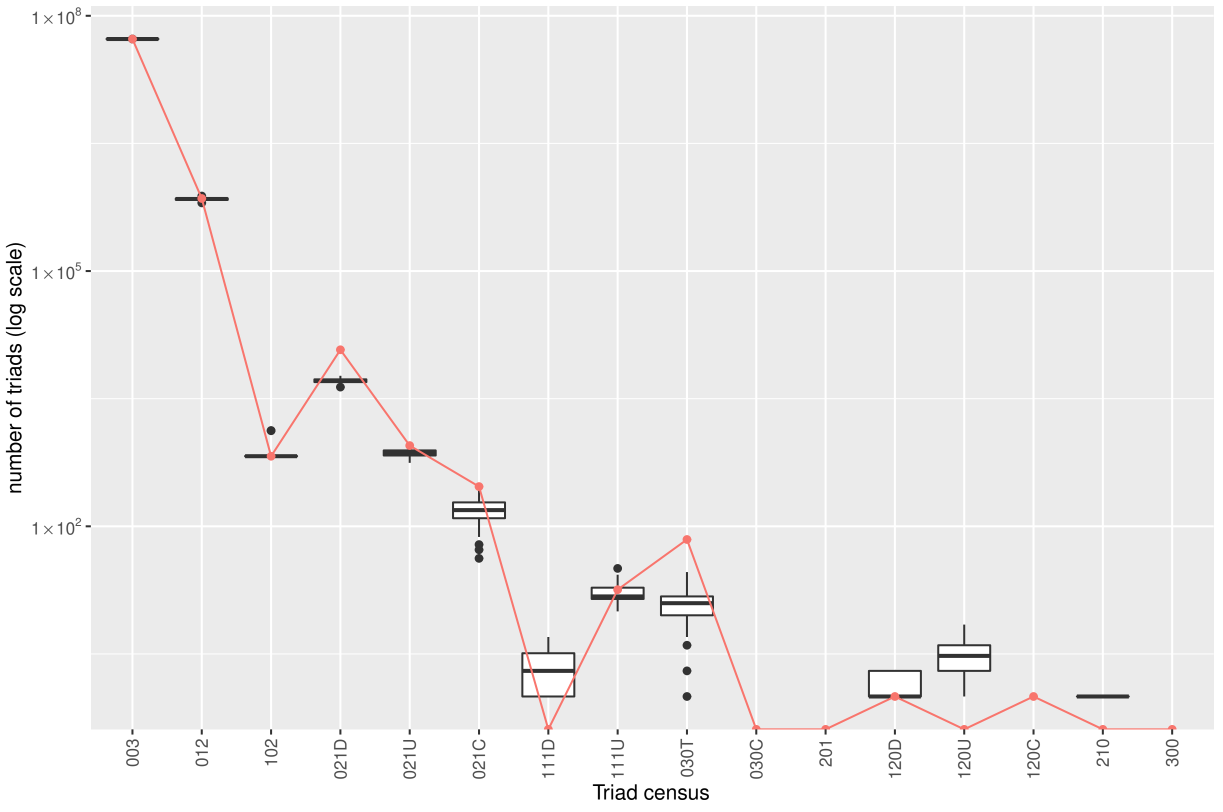

b

Figure S3: (**a**) Goodness-of-fit plots, and (**b**) triad census goodness-of-fit plot, for the Alon yeast regulatory network Model 2, with default decay parameter value $\lambda = 2.0$ (Table S2). The observed network statistics are plotted in red with the statistics of 100 simulated networks plotted as black boxplots, and blue on the histograms.

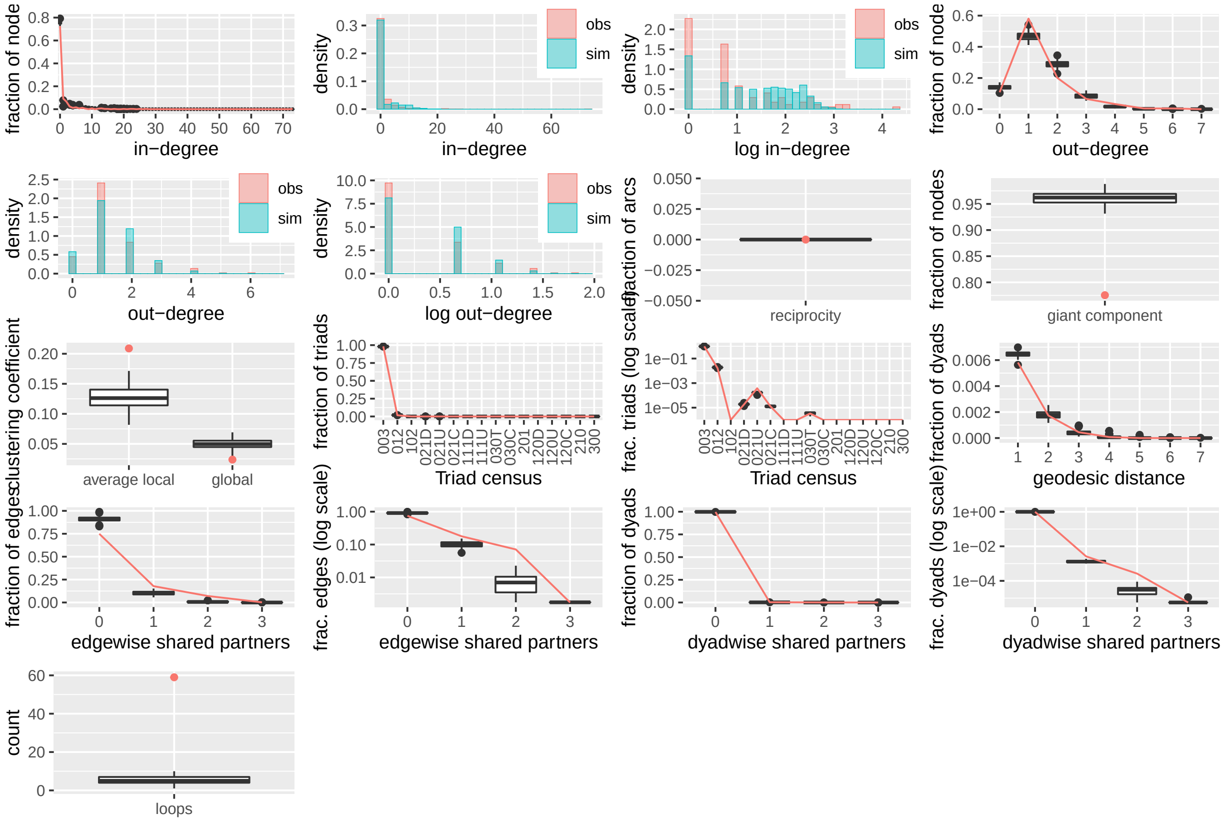

a

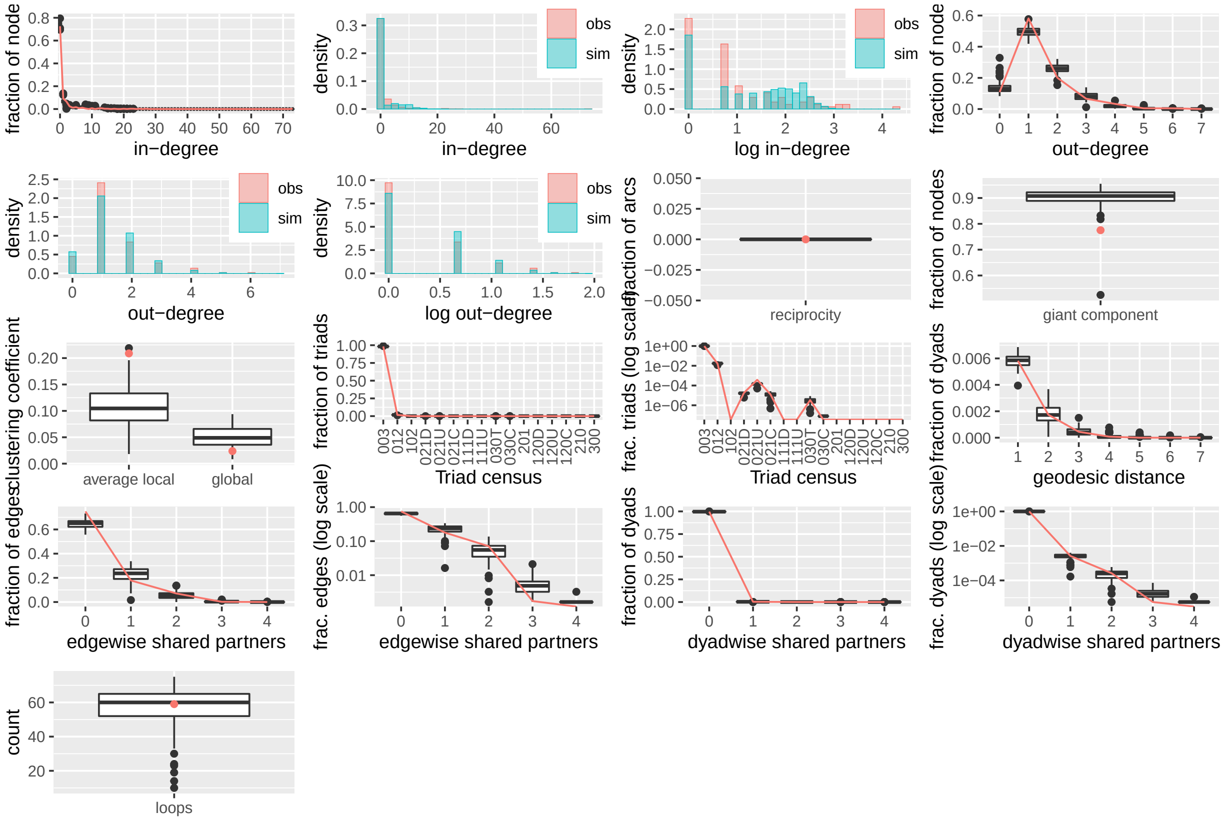

b

Figure S4: Goodness-of-fit plots for the Alon *E. coli* regulatory network with self-edges (Models 3 and 4 in Table 6 in main text). (**a**) Model 3, with no Loop parameter, and (**b**) Model 4, with the Loop parameter included.

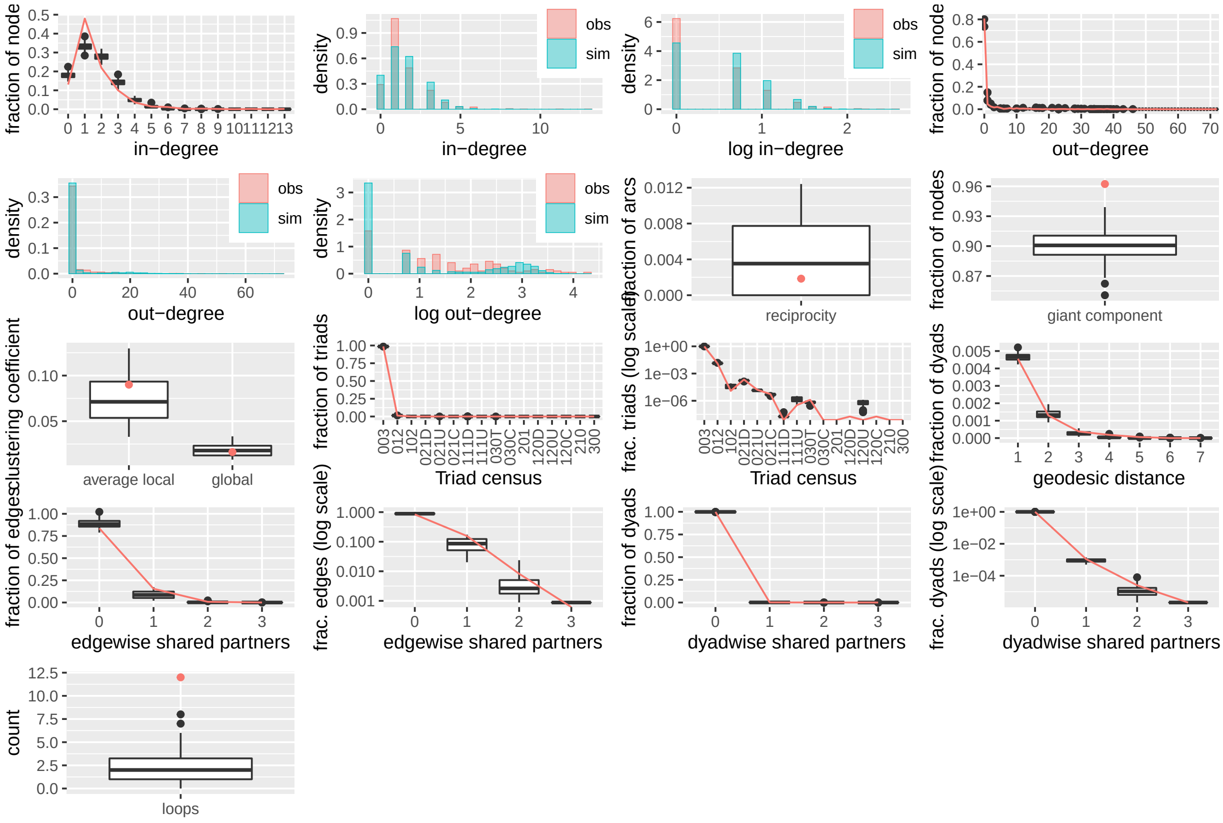

a

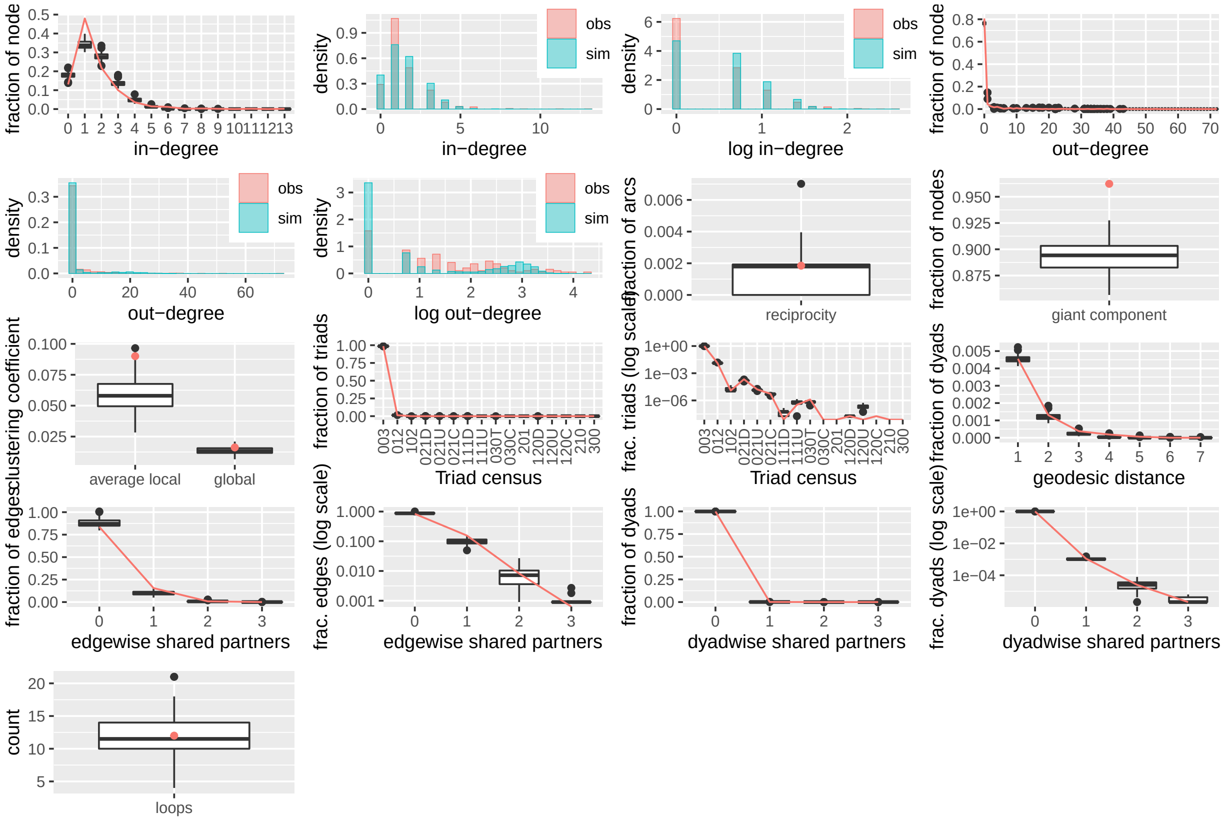

b

Figure S5: Goodness-of-fit plots for the Alon yeast regulatory regulatory network models with self-edges (Table S3). (**a**) Model 1, with no Loop parameter, and (**b**) Model 2, with the Loop parameter included.

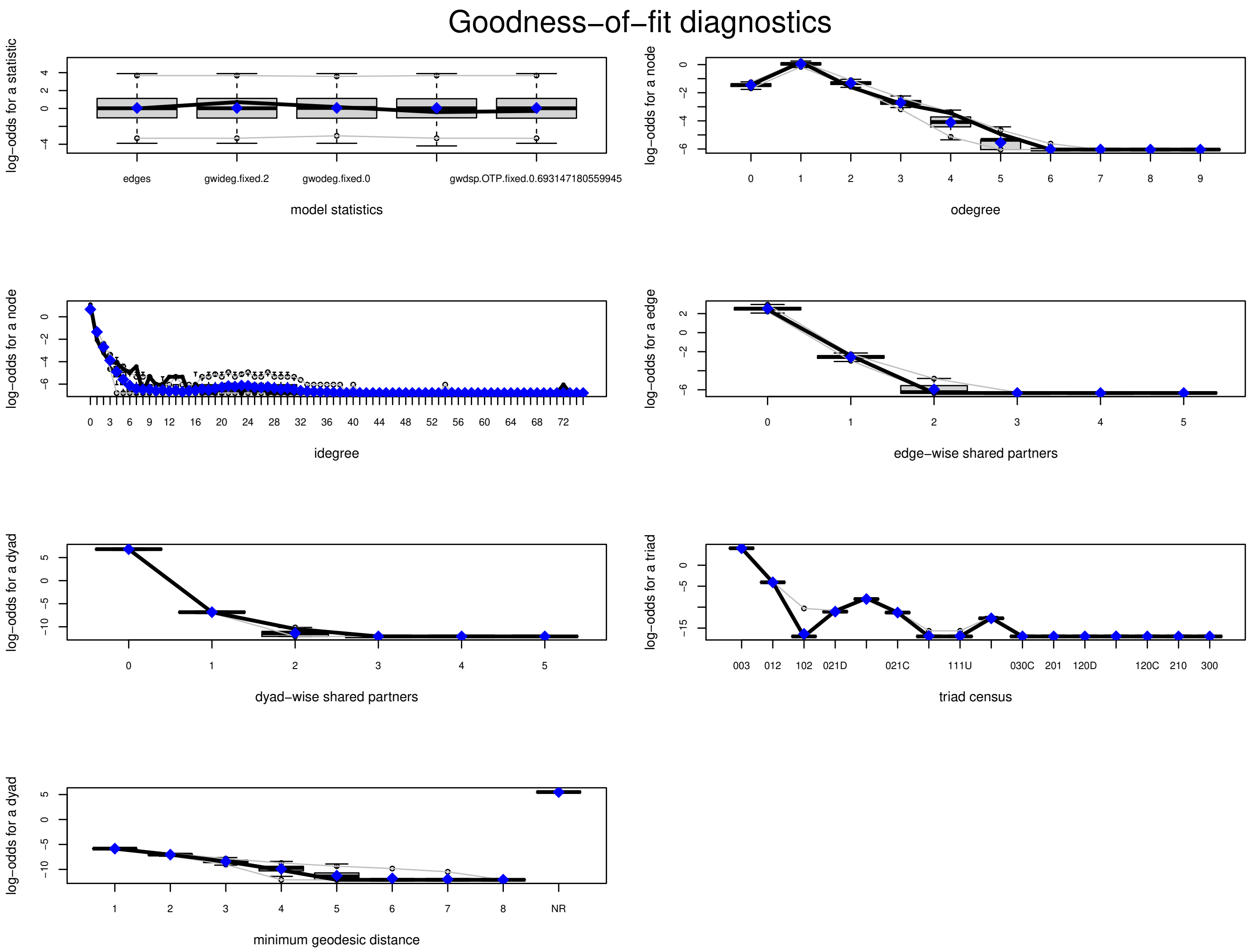

Figure S6: Statnet goodness-of-fit plots for the Alon *E. coli* regulatory network, Model 1 (Table S1).

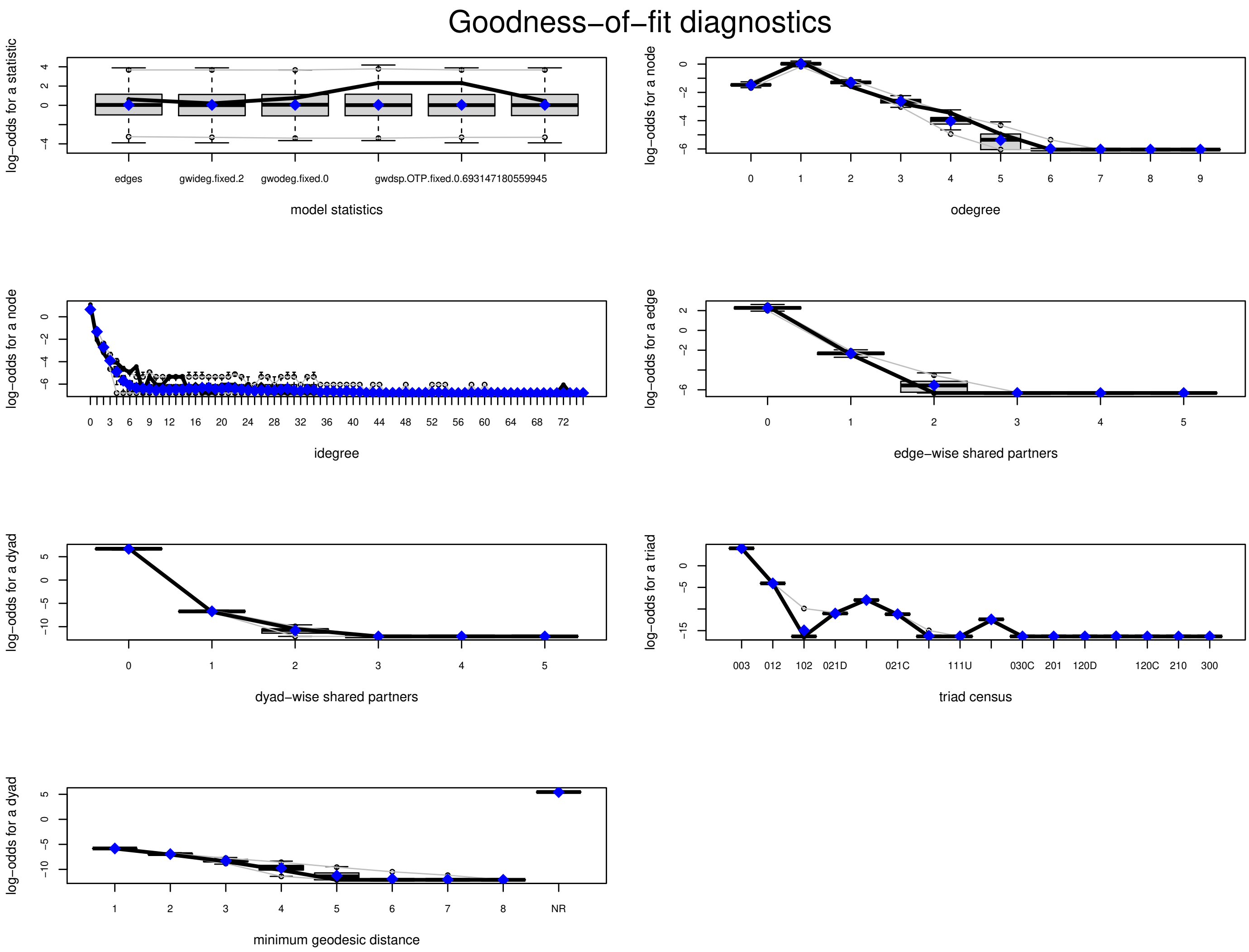

Figure S7: Statnet goodness-of-fit plots for the Alon *E. coli* regulatory network, Model 2 (Table S1).

References

Handcock MS, Hunter DR, Butts CT, Goodreau SM, Morris, Martina (2008) statnet: Software tools for the representation, visualization, analysis and simulation of network data. J Stat Softw 24(1):1–11, URL http://www.jstatsoft.org/v24/i01

Handcock MS, Hunter DR, Butts CT, Goodreau SM, Krivitsky PN, Bender-deMoll S, Morris M (2016) statnet: Software Tools for the Statistical Analysis of Network Data. The Statnet Project (http://www.statnet.org), URL CRAN.R-project.org/package=statnet, R package version 2016.9

Handcock MS, Hunter DR, Butts CT, Goodreau SM, Krivitsky PN, Morris M (2021) ergm: Fit, Simulate and Diagnose Exponential-Family Models for Networks. The Statnet Project (https://statnet.org), URL https://CRAN.R-project.org/package=ergm, R package version 4.1.2

Hummel RM, Hunter DR, Handcock MS (2012) Improving simulation-based algorithms for fitting ERGMs. J Comput Graph Stat 21(4):920–939

Hunter DR, Handcock MS, Butts CT, Goodreau SM, Morris M (2008) ergm: A package to fit, simulate and diagnose exponential-family models for networks. J Stat Softw 24(3):1–29, URL https://www.jstatsoft.org/v024/i03

Krivitsky PN, Hunter DR, Morris M, Klumb C (2021) ergm 4.0: New features and improvements. arXiv preprint arXiv:210604997

Morris M, Handcock M, Hunter D (2008) Specification of exponential-family random graph models: Terms and computational aspects. J Stat Softw 24(4):1–24, URL https://www.jstatsoft.org/v024/i04
